# High-Strength 3D-Ordered Ceramic-Gel Composite Electrolytes Enable Highly Stable Sodium Metal Batteries at − 20 to 60 °C

**DOI:** 10.1007/s40820-025-02032-4

**Published:** 2026-01-04

**Authors:** Liying Shen, Chuyan Hu, Zhenhui Huang, Jiarui Yang, Yanwei Jia, Yufeng Zhao, Rüdiger Berger, Qiang Liu, Yu Zhou

**Affiliations:** 1 State Key Laboratory of Precision Welding & Joining of Materials and Structures, School of Materials Science and Engineering, Harbin Institute of Technology, Harbin, 150001 People’s Republic of China; 2 Max Planck Institute for Polymer Research, 55122 Mainz, Germany; 3 College of Sciences and Institute for Sustainable Energy, Shanghai University, Shanghai, 200444 People’s Republic of China; 4 Institute for Advanced Ceramics, Key Laboratory of Advanced Structural-Functional Integration Materials & Green Manufacturing Technology, School of Materials Science and Engineering, Harbin Institute of Technology, Harbin, 150001 People’s Republic of China

**Keywords:** Ceramic-gel electrolyte, Sodium metal batteries, 3D-Na_3_Zr_2_Si_2_PO_12_ framework, Compressive strength, Flame retardancy

## Abstract

Ceramic-gel composite electrolytes (CGEs) attract significant attention as solid-state electrolytes (SSEs) for sodium metal batteries owing to their favorable ionic conductivity and interfacial compatibility. However, conventional CGEs generally feature insufficient mechanical strength and consequent uncontrollable dendrite growth, remaining long-standing fundamental challenges that severely limit practical applications. Herein, this study presents a high-strength CGE that enables efficient stress transfer, achieving a compressive strength of 20.1 MPa (20 times higher than conventional gel electrolytes), while maintaining excellent ionic conductivity and effectively suppressing sodium dendrites. The 3D-Na_3_Zr_2_Si_2_PO_12_ framework further serves as a thermal barrier, imparting the CGE with superior flame retardancy. Additionally, Na/CGE/NVP-K_0.05_ cells exhibit 75.9% capacity retention after 10,000 cycles at 5C (25 °C) and deliver 78.5 mAh g^−1^ at 30C (60 °C). Remarkably, the CGE exhibits excellent low-temperature adaptability, retaining nearly 100% capacity at –20 °C. These results highlight a viable strategy for designing safe and high-performance solid-state sodium metal batteries toward practical deployment.

**Supplementary Information:**

The online version contains supplementary material available at 10.1007/s40820-025-02032-4.

## Highlights

- A high-strength ceramic-gel electrolyte enables efficient stress transfer, achieving a compressive strength of 20.1 MPa (20 times that of conventional gel electrolytes) while maintaining excellent ionic conductivity and effectively suppressing sodium dendrite growth.
- The Na_3_Zr_2_Si_2_PO_12_ framework acts as a thermal barrier, imparting the ceramic-gel composite electrolytes with superior flame retardancy and maintaining structural integrity after 30 s of burning.
- The structural–functional integration ensures efficient Na⁺ conduction (3.37 × 10^−3^ S cm^−1^) and stable performance from − 20 to 60 °C.

**Supplementary Information:**

The online version contains supplementary material available at 10.1007/s40820-025-02032-4.

## Graphical abstract

**Figure:**
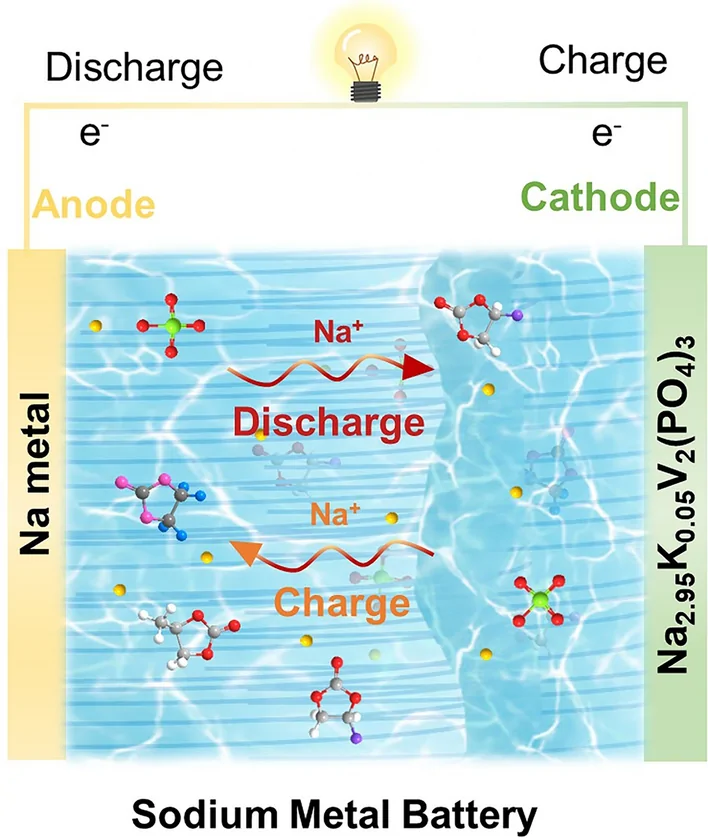

## Introduction

Sodium metal batteries, which utilize sodium metal anodes with a low redox potential (–2.71 V vs. standard hydrogen electrode) and a high theoretical capacity (1165 mAh g^–1^), have emerged as promising candidates for next-generation rechargeable batteries and potential alternatives to lithium-ion batteries [1–5]. Liquid sodium metal batteries have been widely investigated due to their high ionic conductivity (> 10^–3^ S cm^–1^) and favorable electrode/electrolyte interfaces [6–12]. However, the use of conventional organic liquid electrolytes presents significant safety hazards stemming from their flammability, volatility, and potential for leakage. Moreover, these electrolytes often facilitate dendrite growth during cycling, resulting in short circuits and decreased cycle stability.

Solid-state electrolytes (SSEs) have been actively explored to overcome these issues by offering improved safety, better thermal stability, and resistance to dendrite propagation [13–19]. Among various SSE systems, gel polymer electrolytes (GPEs), comprising polymer networks that encapsulate liquid electrolyte components to form a stable gel matrix [20–23], have shown promise due to their relatively high ionic conductivity and enhanced interfacial contact with electrodes. The incorporation of liquid phases enables Na^+^ transport via polymer segmental motion and diffusion within the swollen gel or liquid domains, achieving ionic conductivities and interfacial properties comparable to those of liquid electrolytes [24]. Additionally, the highly cross-linked polymer framework imparts flexibility while effectively minimizing the risk of liquid leakage [25]. Nevertheless, their inherent mechanical weakness and insufficient thermal stability remain bottlenecks, limiting their application in metal batteries [26].

**Fig. 1:**
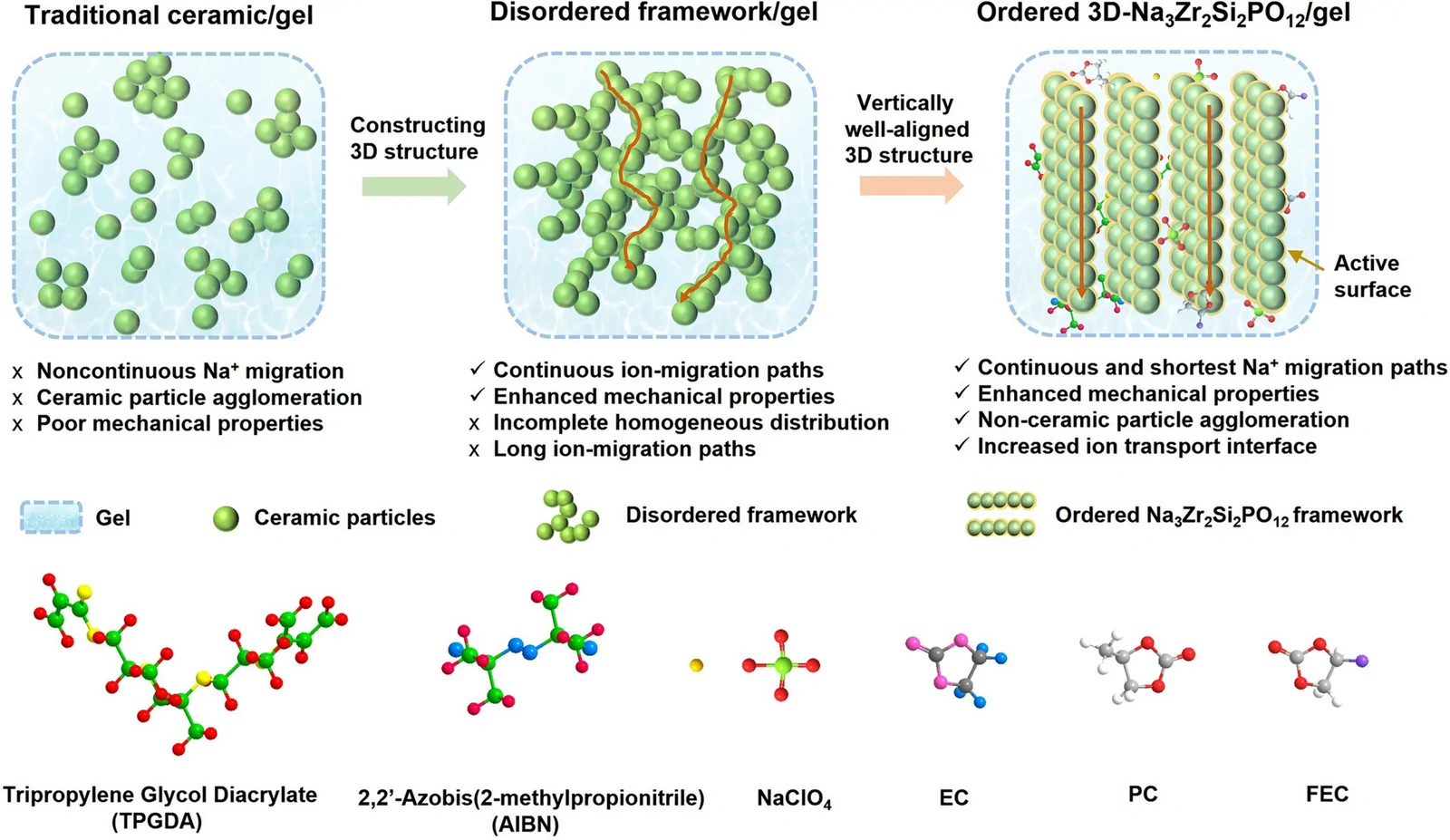
Comparison of conventional composite gel electrolytes and disordered and ordered three-dimensional framework-filled composite gel electrolytes

To address these challenges, ceramic-gel composite electrolytes (CGEs) are expected to address the poor thermal stability and dendrite growth issues of GPEs [27–30]. To this end, non-conductive fillers such as SiO_2_ [31] and commercial glass fiber (GF) membranes [27] have been embedded to enhance the dimensional stability of GPEs. The SiO₂-reinforced composite gel electrolytes developed by Yin et al. [32] combine high mechanical strength (hardness 0.41 GPa), effectively suppressing dendrite penetration and display a capacity retention of 98% at 0.2C after 400 cycles. However, these materials often introduce undesirable interfacial resistance due to their non-ionic nature, limiting their effectiveness in facilitating uniform Na plating/stripping. Incorporating ionically conductive Li_6.5_La_3_Zr_1.5_Ta_0.5_O_12_ (LLZTO) particles into a polymer gel, Wu et al. [33] obtained a composite gel electrolyte with a tensile strength of 9.9 MPa, albeit with a reduced ionic conductivity (7.89 × 10⁻^4^ S cm⁻^1^). Lei et al. [34] employed an ionically conductive ceramic framework (β/β″-Al₂O₃) to establish a continuous Na⁺-transport network that enhances the mechanical strength of the gel electrolyte, suppresses dendrite growth, and enables uniform sodium deposition. As a result, the NVP/ANs–GPE/Na cell demonstrates excellent capacity retention of 78.8% after 1000 cycles at 1C and 60 °C. Despite these advances, the random dispersion of ceramic particles or disordered ceramic framework within composite gel electrolytes often leads to localized stress concentrations rather than uniform load distribution, resulting in mechanically weak structures (Fig. 1). Consequently, the insufficient mechanical strength of CGEs and the consequent uncontrollable dendrite growth remain long-standing fundamental challenges that severely limit practical applications. Therefore, developing a homogeneous inorganic ionic conductor/gel composite with a compact structure is crucial for enhancing the mechanical strength and battery stability of ceramic-gel composite electrolytes.

**Fig. 2:**
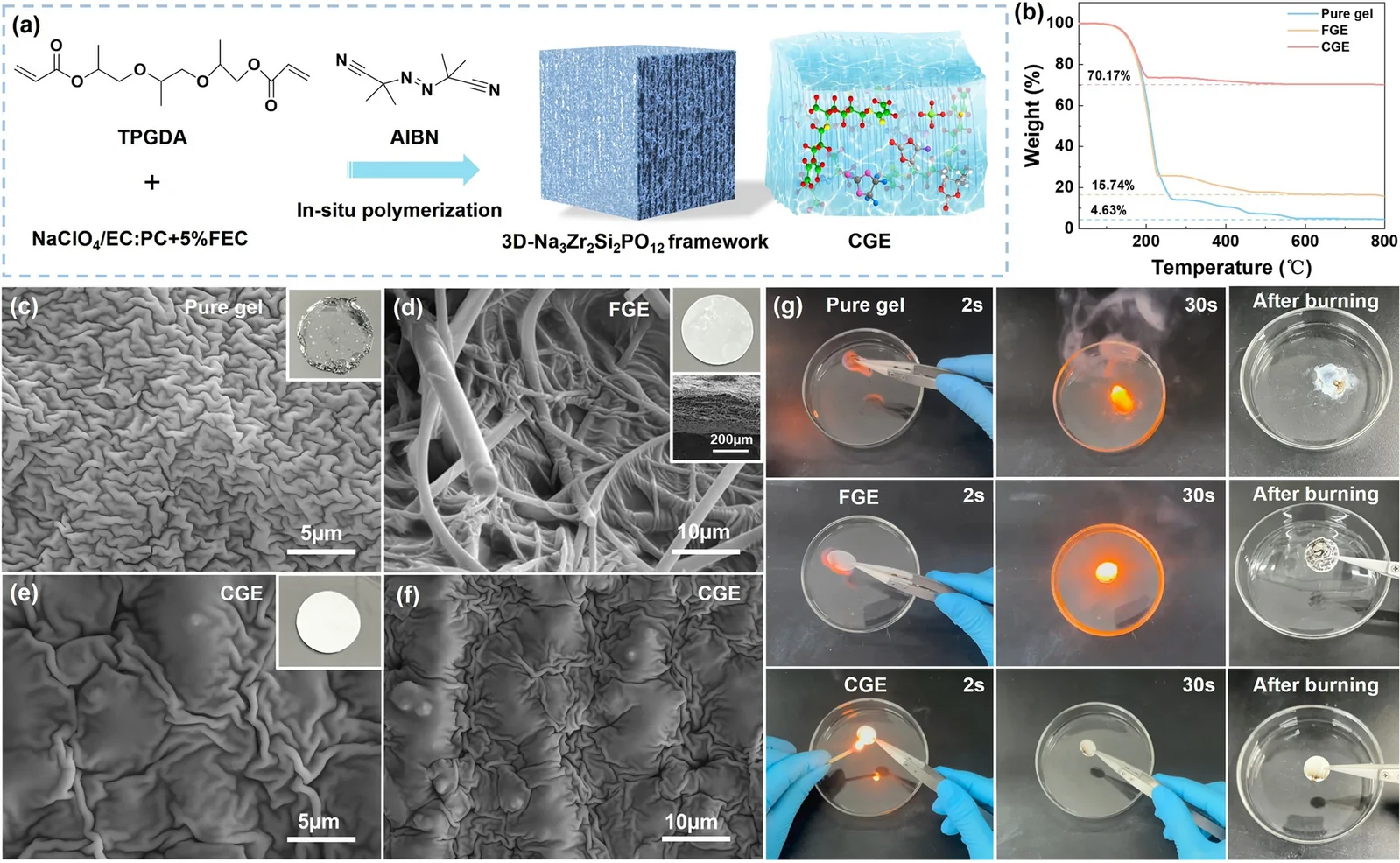
Design strategy, structure, and thermal stability of CGE. **a** Schematic diagram of composite gel electrolyte synthesis, **b** TGA curves of the pure gel, FGE and CGE. SEM images: **c** pure gel, **d** FGE and **e**, **f** CGE, **g** Flammability test of pure gel, FGE and CGE

In this work, we report a high-strength ceramic-gel composite electrolyte. The core structural design of this composite electrolyte is based on a three-dimensional (3D) vertically aligned Na_3_Zr_2_Si_2_PO_12_ framework. Notably, the 3D ceramic framework exhibits a highly ordered vertical alignment characteristic. This regular spatial structure not only further enhances the mechanical support effect but also guides the directional transport of sodium ions along the vertical direction. Meanwhile, a pore structure with uniform size and regular distribution is formed between the frameworks. This pore structure also provides sufficient space for the filling of the gel phase, as shown in Fig. S1. The framework is infiltrated with an organic gel electrolyte precursor that fills its ordered channels (Fig. 2a). The vertical alignment of the framework prevents inorganic filler agglomeration and mitigates stress concentration caused by the disordered ceramic framework. This architecture enables efficient stress transfer in the 3D-Na_3_Zr_2_Si_2_PO_12_/gel CGE, achieving a compressive strength of 20.1 MPa (20 times higher than conventional gel electrolytes), while maintaining excellent ionic conductivity (3.37 × 10⁻^3^ S cm⁻^1^ at room temperature, RT) and effectively suppressing sodium dendrites growth. The Na₃Zr₂Si₂PO₁₂ framework also acts as a thermal barrier, endowing the CGE with superior flame retardancy. Additionally, the structural–functional integration strategy delivers efficient Na⁺ conduction and overcomes temperature sensitivity, ensuring stable performance from − 20 to 60 °C. These results highlight a viable strategy for designing safe and high-performance solid-state sodium metal batteries toward practical deployment.

## Experimental Sections

### Materials

All chemical reagents used in material preparation were of analytical grade, including Na_2_CO_3_ (≥ 99.0%, Aladdin), NH_4_H_2_PO_4_ (≥ 99.0%, Aladdin), SiO_2_ (≥ 99.99%, Aladdin), ZrO_2_ (≥ 99. 99%, Aladdin), anhydrous acetonitrile (≥ 99.8%, Aladdin), NaH_2_PO_4_ (≥ 99.0%, Aladdin), KH_2_PO_4_ (≥ 99.0%, Aladdin), NH_4_VO_3_ (≥ 99.0%, Aladdin), glycolic acid (≥ 99.0%, Aladdin), tripropylene glycol diacrylate (TPGDA, ≥ 90.0%, Aladdin), 2,2’-azobis(2-methylpropionitrile) (AIBN, ≥ 99.0%, Aladdin), and NaClO_4_/EC:PC + 5%FEC (Guangdong Canrd New Energy Technology Co., Ltd.).

### Synthesis of Composite Gel Electrolyte

#### ***Preparation of the 3D-Na***_***3***_***Zr***_***2***_***Si***_***2***_***PO***_***12***_*** Inorganic Framework (3D/ISE)***

Stoichiometric amounts of precursors, including Na_2_CO_3_, NH_4_H_2_PO_4_, SiO_2,_ and ZrO_2_, were mixed with anhydrous ethanol via a mixing machine for 12 h. To compensate for the loss of volatility during sintering, a 10 wt% excess of Na or P was added to the original compound. The obtained Na_3_Zr_2_Si_2_PO_12_ precursor powders were heated at 80 °C overnight to remove the anhydrous ethanol. The samples were then sintered at 1,100 °C for 3 h at a rate of 3 °C min^−1^ in a muffle furnace. After sintering, the Na_3_Zr_2_Si_2_PO_12_ powders were ball-milled for 24 h.

The ordered Na_3_Zr_2_Si_2_PO_12_ inorganic framework was fabricated using a freeze-drying technique. Initially, the pre-synthesized Na_3_Zr_2_Si_2_PO_12_ powder was dispersed to form an aqueous slurry with a volume fraction of 25%. The selection of the molds and the demolding method is critical for the forming and maintaining of the final material structure. This study employed cylindrical polypropylene molds (diameter ∼25 mm, height 4 cm) to ensure uniform samples morphology. To facilitate subsequent demolding, a thin layer of Vaseline was uniformly applied to the inner wall of the molds as a release agent before freezing. Subsequently, the degassed ceramic slurry was slowly injected into the molds and rapidly frozen on a copper plate cooled by liquid nitrogen. After the samples were completely solidified at -196 °C, the demolding operation was performed. With the assistance of Vaseline’s lubricating properties, the intact frozen samples could be smoothly released by gently pressing the bottom of the molds. Finally, the samples were promptly transferred to a freeze-dryer and continuously dried at − 40 °C for 72 h to completely remove the solvent, ultimately obtaining a porous Na_3_Zr_2_Si_2_PO_12_ green body with a three-dimensionally ordered structure. The resulting structure was then sintered at 650 °C for 2 h with a heating rate of 2 °C min^−1^, followed by a second sintering step at 1,200 °C for 5 h at a heating rate of 3 °C min^−1^, yielding an ordered 3D-Na_3_Zr_2_Si_2_PO_12_ inorganic framework.

The disordered Na_3_Zr_2_Si_2_PO_12_ inorganic framework was synthesized via a sacrificial template method. Specifically, Na_3_Zr_2_Si_2_PO_12_ powder was mixed with 20 μm diameter poly(methyl methacrylate) (PMMA) microspheres at a defined volume ratio. The mixture was uniaxially pressed into 18 mm diameter pellets under 3 MPa. To remove the PMMA template, the green bodies were first heat-treated at 700 °C for 3 h in a muffle furnace. Subsequently, the pellets were sintered at 1,200 °C for 5 h using a controlled heating program: 2 °C min⁻^1^ to 650 °C, then 3 °C min⁻^1^ to 1,200 °C. The resulting sample with a disordered porous structure was obtained.

#### ***Preparation of the 3D-Na***_***3***_***Zr***_***2***_***Si***_***2***_***PO***_***12***_*** Composite Gel Electrolyte***

The ordered 3D-Na_3_Zr_2_Si_2_PO_12_ CGE was prepared via in situ polymerization of a precursor within the 3D-Na_3_Zr_2_Si_2_PO_12_ framework. To evaluate the impact of thickness, the Na_3_Zr_2_Si_2_PO_12_ frameworks were fabricated via a standardized wire-cutting process with this parameter precisely controlled at two distinct values: ~ 0.6 mm and ~ 1.35 mm. The precursor solution comprised 2 wt% TPGDA (C_15_H_24_O_6_) monomer and 0.1 wt% AIBN (C_8_H_12_N_4_) initiator and was dissolved in a liquid electrolyte comprising 1 M NaClO_4_ in EC/PC with 5% FEC. The 3D-Na_3_Zr_2_Si_2_PO_12_ framework was immersed in the gel precursor solution. A key step involved placing the entire setup under vacuum to evacuate air from the pores, thereby enabling the precursor solution to infiltrate the framework’s entire porous network completely. Subsequently, the infiltrated framework was subjected to an in situ curing process inside an argon-filled glovebox to form the composite gel electrolyte. The products from the ordered and disordered frameworks are denoted as CGE and D-CGE, respectively.

#### Preparation of the Glass Fiber Composite Gel Electrolyte (FGE)

The FGE was prepared through in situ polymerization of a precursor solution infused into the pores of a Whatman GF glass fiber membrane. The composition of the gel precursor solution and the polymerization procedure were identical to those used for the fabrication of the composite gel electrolyte.

#### ***Synthesis of the Na***_***2.95***_***K***_***0.05***_***V***_***2***_***(PO***_***4***_***)***_***3***_*** Electrode Material (NVP-K***_***0.05***_***)***

The Na_2.95_K_0.05_V_2_(PO_4_)_3_ material was synthesized via a sol–gel method involving Na_2_CO_3_ (≥ 99.0%), NaH_2_PO_4_ (≥ 99.0%), KH_2_PO_4_ (≥ 99.0%), NH_4_VO_3_ (≥ 99.0%), and glycolic acid as precursors. The detailed synthesis procedure was described in our previous study [35].

### Material Characterization

X-ray diffraction (XRD, Empyrean, Panalytical BV, Almelo, The Netherlands) was performed using Cu-Kα radiation to determine the crystalline phases. The morphology and elemental distribution were examined via field-emission scanning electron microscopy (FESEM, Hitachi, SU5000, Japan) equipped with energy-dispersive X-ray spectroscopy (EDS). Quasistatic uniaxial compression tests were conducted via a universal testing machine (Instron 5569, Instron Co., Canton, USA) at a strain rate of 10^–3^ s^–1^ with cylindrical samples (Φ 5 mm × 10 mm). Fourier transform infrared spectroscopy (FT-IR, Nicolet iS50) was employed to analyze the chemical structure of the samples. Thermal behavior was characterized using thermogravimetric analysis (TG-DSC, NETZSCH, STA449F3, Germany) at a heating rate of 10 °C min^–1^ under a nitrogen atmosphere. The chemical states of element were researched using X-ray photoelectron spectroscopy (XPS, PHI-QUANTERA-II-SXM).

### Electrochemical Characterization

Electrochemical impedance spectroscopy (EIS) measurements were conducted via a CHI760 electrochemical workstation over a frequency range of 1 MHz to 0.01 Hz.

The ionic conductivity (σ) was calculated via the following equation:

1 $$\sigma =\frac{L}{RS}$$

where *σ* is the ionic conductivity, *L* is the electrolyte thickness, and *R* and *S* represent the electrolyte resistance and the area of the sample, respectively.

The electrochemical stability window of the electrolytes was evaluated via linear sweep voltammetry (LSV) via Na/electrolytes/SS cells over a voltage range of 2.5–6.5 V at a scanning rate of 0.1 mV s^−1^ on a CHI760 workstation. The cathode was fabricated by combining NVP-K_0.05_, Super P, and polyvinylidene fluoride (PVDF) in a weight ratio of 75:15:10, using N-methyl-2-pyrrolidone as the dispersing solvent. After drying at 80 °C, the cathodes with an NVP-K_0.05_ loading of 2–3 mg were obtained. The electrochemical performance of the CR2032 coin cells was evaluated with a Land testing system (LAND CT3002AU). All assembly processes were carried out in an argon-filled glove box (O_2_ ≤ 0.1 ppm, H_2_O ≤ 0.1 ppm) to prevent environmental contamination. Galvanostatic charge–discharge testing was conducted within a voltage window of 2.3–3.9 V. The NVP-K_0.05_/electrolyte/Na cells were tested over a temperature range of –20 to 60 °C in temperature-controlled ovens. For Na/Na symmetric cells, charge–discharge cycling was performed with 2 h steps at varying current densities.

### First-Principles Calculations

First-principles calculations were performed using the Cambridge Serial Total Energy Package. The two-dimensional Na_3_Zr_2_Si_2_PO_12_ slab was constructed by cleaving the [001] direction of the Na_3_Zr_2_Si_2_PO_12_ conventional cell with a 2 × 2 supercell configuration. The 2D crystal model of Na_3_Zr_2_Si_2_PO_12_ was relaxed using density functional theory (DFT) with the Perdew–Burke–Ernzerhof (PBE) functional. A vacuum layer of 2 nm was introduced between adjacent repeating units of the 2D slabs to avoid interlayer interactions. All atomic positions within the constructed 2D slabs were fully relaxed. Ultrasoft pseudopotentials are applied to describe the ionic cores with a plane-wave cutoff energy of 370 eV. The k-point sampling grids are 3 × 3 × 1 for 2D crystal slabs. The Na-ion diffusion routes at the inner channel (A route) and surface channel (B route) are shown, with a step size of 0.05 in fractional coordinates.

## Results and Discussion

### Synthesis and Characterization

We first investigated gel formation in the framework. FTIR was employed to investigate the polymerization process of TPGDA in NaClO_4_/EC:PC with 5% FEC electrolyte (Fig. S2). The characteristic C = C stretching vibration at 1620 cm^–1^, attributed to TPGDA, was nearly absent following polymerization, confirming efficient monomer conversion (Fig. S3) [36]. Figure 2b presents the TGA curves for the pure gel, the glass FGE used as a reference, and the CGE. All samples demonstrated minimal weight loss below 100 °C, suggesting that the framework effectively confines the gel/liquid components, thereby inhibiting their evaporation under elevated temperature conditions. Upon heating to 270 °C, rapid weight loss occurred in all the samples, which was attributed to the decomposition or volatilization of the liquid components. At 600 °C, the pure gel and FGE resulted in weight losses of 95.37% and 84.26%, respectively, whereas the CGE resulted in a significantly lower weight loss of only 29.83%, indicating that about 31.29 wt% of the gel-filled the Na_3_Zr_2_Si_2_PO_12_ framework.

The weight percentage (*X*) of the gel component was calculated using the following equation:

2 $$X=\frac{{W}_{CGE}}{{W}_{Gel}}$$

where *X* is the weight percentage of gel in the composite gel electrolyte, *W*_*CGE*_ is the weight loss of the composite gel electrolyte, and *W*_*Gel*_ is the weight loss of the pure gel. Since the composite electrolyte primarily consists of Na_3_Zr_2_Si_2_PO_12_ ceramics, it demonstrates enhanced thermal stability and heat resistance [37]. Complementary to the thermal stability, the microstructures of the electrolytes were examined. Figure 2c–f shows the microstructures of the pure gel, FGE, and CGE, with the inset showing a photo of the electrolyte. The pure gel exhibited a wrinkled surface morphology resulting from high-temperature evaporation during gold sputtering (Fig. 2c). The cross-sectional morphology of the commercial glass fiber (GF) used as a reference structural separator combined with the gel exhibited good integration, with the gel fully penetrating the randomly arranged fiber rods, as observed in Fig. 2d and further confirmed by the EDS mapping in Fig. S4. Figure 2e, f illustrates that the gel precursor solution completely infiltrates the 3D-Na_3_Zr_2_Si_2_PO_12_ framework under vacuum conditions, forming a robust interfacial contact with the solid framework. The distribution of Na, Zr, Si, P, O, C, F, and Cl in the EDS mapping further confirmed the uniform distribution of the gel and its strong interaction with the Na_3_Zr_2_Si_2_PO_12_ framework (Fig. S5). Flame tests revealed that both the pure gel and FGE ignited immediately upon contact with an open flame and were almost entirely consumed, leaving minimal residue. In contrast, the CGE, reinforced with the 3D-Na_3_Zr_2_Si_2_PO_12_ framework, demonstrated enhanced flame retardancy. When exposed to a direct flame for 2 s, CGE resisted ignition. After 30 s, while the pure gel and FGE continued to burn intensely. In contrast, the CGE showed excellent flame retardancy and preserved its structural integrity (Fig. 2g, Videos S1-S3). This superior flame resistance is primarily attributed to the high content of the non-combustible Na_3_Zr_2_Si_2_PO_12_ framework within the composite gel electrolyte, effectively reducing safety hazards.

### Physicochemical Properties and High Temperature Electrochemical Performance

**Fig. 3:**
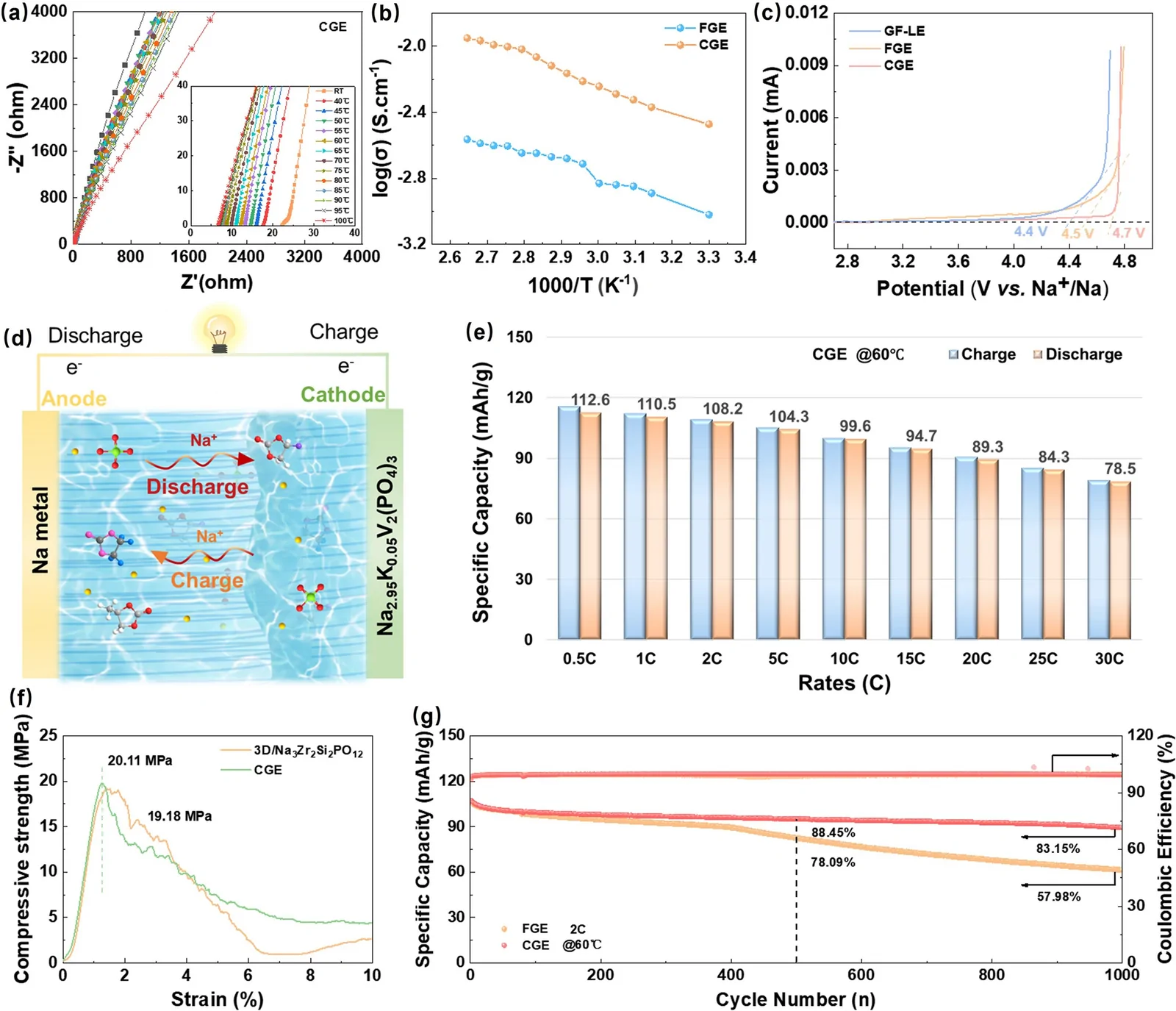
**a** Nyquist plots of the CGE with a thickness of 1.35 mm and a diameter of 15 mm, **b** Arrhenius plots of the ionic conductivity of the FGE and CGE at various temperatures, **c** LSV curves of the GF-LE, FGE, and CGE, **d** Schematic diagram of solid-state sodium batteries assembled with the CGE and Na_2.95_K_0.05_V_2_(PO_4_)_3_ (NVP-K_0.05_) cathode, **e** Rate capability of the Na/CGE/NVP-K_0.05_ full cell from 0.5C to 30C, **f** Compressive strength–strain curves of the 3D-Na_3_Zr_2_Si_2_PO_12_ framework and CGE, **g** Long-term cycling performance of the Na/NVP-K_0.05_ full cell

EIS was employed to analyze the charge transport dynamics at the electrode/electrolyte interface for both FGE and CGE across a temperature range from RT to 100 °C (Figs. 3a and S6). The Nyquist plots reveal distinct impedance profiles for CGE and FGE, reflecting their temperature-dependent transport behaviors (Fig. 3b). At RT, the CGE demonstrates a high ionic conductivity of 3.37 × 10^–3^ S cm^–1^, corresponding to roughly 50% of the conductivity observed for the liquid NaClO_4_/EC:PC with 5% FEC electrolyte (7.86 × 10^–3^ S cm^–1^). Notably, this performance is approximately 3 times superior to that of the FGE (~ 1.0 × 10⁻^3^ S cm⁻^1^) and over 10 times higher than that of the 3D-Na₃Zr₂Si₂PO₁₂ framework (3.31 × 10⁻^4^ S cm⁻^1^), as detailed in Fig. S7, and Table S1. These results indicate that gel‑phase incorporation significantly enhances the ion‑transport efficiency within the ordered Na_3_Zr_2_Si_2_PO_12_ framework compared with the non-ionic conductive disordered framework, improving the ionic conductivity across various temperatures. The integration of inorganic ceramics reportedly contributes to the increased oxidative decomposition resistance of the composite electrolyte [38]. To verify this, we evaluated the electrochemical stability of the composite gel electrolyte via linear sweep voltammetry (LSV). The decomposition voltages of the glass fibers with the liquid electrolyte (GF-LE) and FGE are 4.4 and 4.5 V, respectively (Fig. 3c). Notably, the introduction of a 3D-Na_3_Zr_2_Si_2_PO_12_ ceramic framework into the gel electrolyte increased the decomposition voltage to 4.7 V. Given the favorable ionic conductivity and electrochemical stability of the CGE, it was integrated into a practical Na/NVP-K_0.05_ sodium metal battery to evaluate its practical applicability (Fig. 3d). The Na/CGE/NVP-K_0.05_ battery exhibits specific discharge capacities of 112.6, 110.5, 108.2, 99.6, 94.7, 89.3, 84.3, and 78.5 mAh g^–1^ from 0.5C to 30C at 60 °C (Figs. 3e and S8), reflecting the efficient sodium-ion transport performance of CGE. The limited mechanical properties of gel electrolytes are well recognized as a major obstacle to their practical application in energy storage devices. In sharp contrast, the 3D-Na_3_Zr_2_Si_2_PO_12_-based composite gel electrolyte demonstrates outstanding mechanical robustness, achieving a compressive strength of 20.11 MPa (Fig. 3f), which is more than an order of magnitude higher than that of conventional composite gel electrolytes (~ 1 MPa) [39]. Under compressive loading, this material not only preserves excellent structural integrity but also benefits from its ordered porous architecture, which ensures uniform stress distribution and mitigates local stress concentration, thereby delivering superior mechanical performance. Nevertheless, gel electrolytes typically face the inherent trade-off between mechanical strength and ionic conductivity (Table S2) [33, 40–48]. Pristine gels lack sufficient mechanical robustness for practical application. To address this, the composite integrated with a 3D-ordered ceramic framework markedly enhances the overall load-bearing capacity and imparts greater inelastic deformation capability compared with composites reinforced with randomly dispersed particles or disordered non-ionic conductive networks [49]. As a result, the 3D-Na_3_Zr_2_Si_2_PO_12_-based composite gel electrolyte plays a pivotal role in suppressing dendrite growth, maintaining structural stability, and enabling the long-term safe operation of sodium metal batteries. As illustrated in Fig. 3g, the CGE-based full cell exhibited remarkable cycling stability at 60 °C, maintaining 83.15% specific capacity retention after 1,000 cycles, significantly surpassing the retention observed for the FGE-based battery (57.98%). This outstanding performance can be attributed to two synergistic effects: (i) enhanced Na^+^ transport efficiency facilitated by the gel phase, which reduces the overall resistance, and (ii) improved chemical and electrochemical stability provided by the 3D-Na_3_Zr_2_Si_2_PO_12_ framework, which reinforces the structural integrity of the electrolyte.

### Room Temperature Electrochemical Performance

**Fig. 4:**
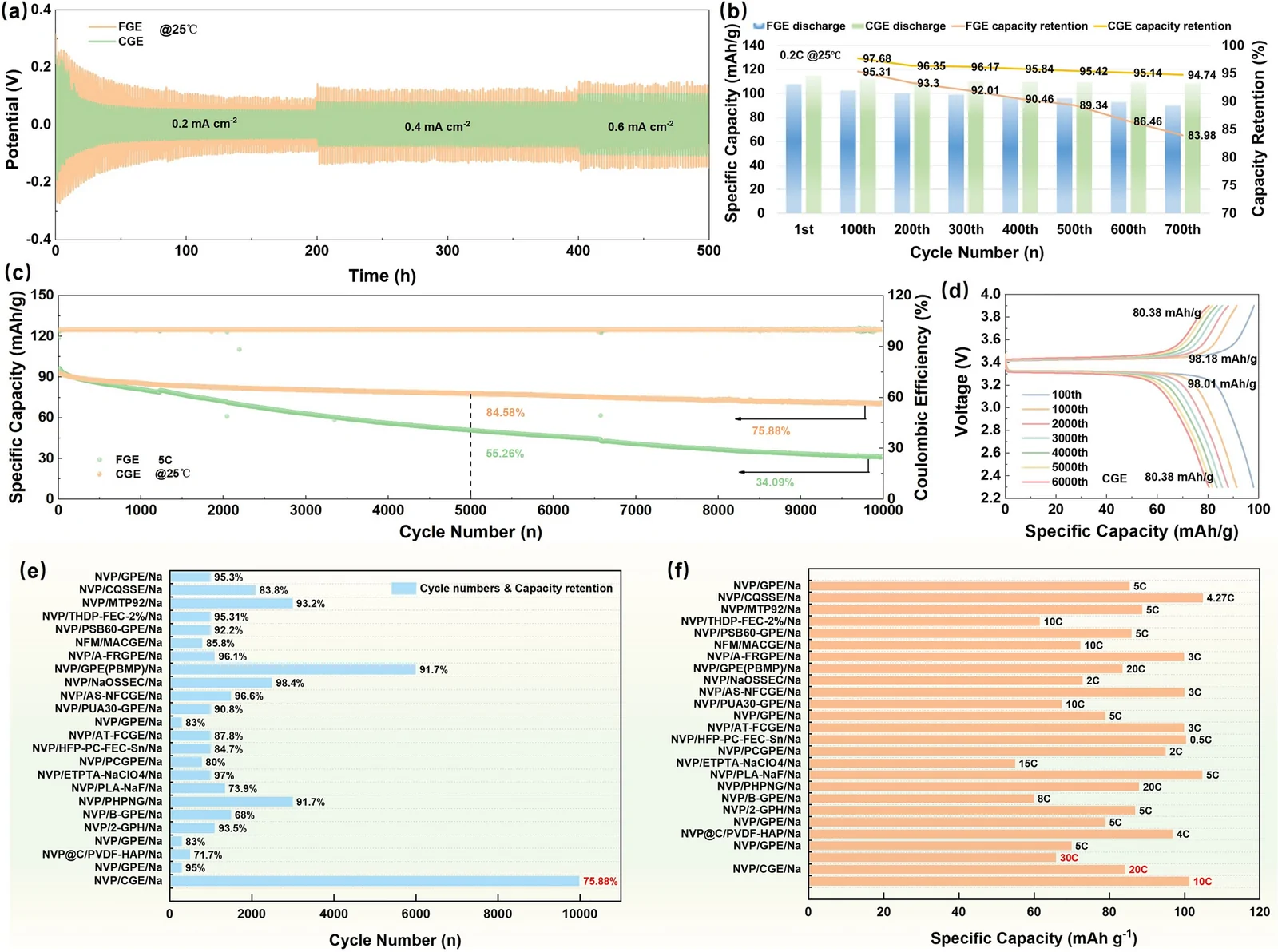
Electrochemical performance tests of FGE and CGE. **a** The polarized voltage measurement of Na/Na symmetric battery from 0.2 to 0.6 mA cm^−2^ at 25 °C, **b** Comparison of the cycling stability of sodium metal batteries at 0.5C, **c** Ultra-long cycling performance at 5C, **d** Charge/discharge profiles for the different cycles of CGE at 2C, **e**, **f** Comparison of the cycling stability and rate performance for sodium full cells with state-of-the-art gel electrolytes

To further verify the effectiveness of the composite gel electrolyte in enhancing electrode/electrolyte interface stability, a systematic electrochemical evaluation was performed on Na/Na symmetric cells and Na/NVP-K_0.05_ full cells at 25 °C. In the Na/Na symmetric configuration, the Na/CGE/Na cell exhibited consistently low and stable polarization voltages at current densities of 0.2, 0.4, and 0.6 mA cm^–2^, indicating lower interfacial resistance and improved compatibility (Fig. 4a). In comparison, the Na/FGE/Na cell showed considerable voltage fluctuations under the same testing conditions, reflecting inferior interfacial stability and further confirming the superiority of the CGE in facilitating electrode/electrolyte interfacial kinetics. To further investigate the interfacial stability, EIS measurements were conducted on a symmetric Na/Na cell employing the CGE at various cycling stages (before cycling, after the 10th cycle, and after the 60th cycle). The evolution of the interfacial resistance revealed a pronounced decline in the early cycling stage, dropping rapidly to below 400 Ω within the first 10 cycles (Fig. S9). Upon extended cycling to 60 cycles, the resistance was reduced by more than an order of magnitude and exhibited stabilization. The continued resistance lowering underscores the dynamic optimization of the electrode/electrolyte interface, which matures into a stable low-impedance phase during repeated sodium stripping/plating. This resistance evolution demonstrates the formation of a highly stable interface in the CGE system, a key factor for long-term cycling durability. In cycling tests of Na/NVP-K_0.05_ cells, the Na/CGE/NVP-K_0.05_ cell retained a discharge capacity of 108.5 mAh g^–1^ after 700 cycles, with a high-capacity retention of 94.74% and an average capacity decay of 0.0075% per cycle (Figs. 4b and S10). In contrast, the Na/FGE/NVP-K_0.05_ cell exhibited a more pronounced capacity decline, with capacity decreasing to 90.2 mAh g^–1^ and a retention of 83.98% after 700 cycles. To evaluate the high-rate cycling stability of the CGE, systematic tests were performed on full cells assembled with various composite gel electrolytes. Notably, the Na/CGE/NVP-K_0.05_ cell demonstrated excellent long-term performance. It achieved capacity retentions of 84.58% after 5,000 cycles and 75.88% after 10,000 cycles, while maintaining a discharge capacity of 70.5 mAh g⁻^1^. This performance was significantly superior to that of the Na/FGE/NVP-K_0.05_ cell, which exhibited capacity retentions of 55.26% after 5000 cycles and 34.09% after 10,000 cycles, with the discharge capacity decreasing to 31.4 mAh g^–1^ (Fig. 4c). Furthermore, the Na/CGE/NVP-K_0.05_ cell demonstrated superior cycling stability at a 2C rate, retaining 78.45% of its initial capacity after 6,907 cycles, far exceeding the 40.59% retention of the Na/FGE/NVP-K_0.05_ cell (Fig. S11). After 6,000 cycles, the CGE system delivered a discharge capacity of 80.38 mAh g⁻^1^, nearly double the 42.45 mAh g⁻^1^ of the FGE system (Figs. 4d and S12). The Na/CGE/NVP-K_0.05_ cell demonstrated exceptional rate capability across various charge/discharge rates. Even at an ultrahigh rate of 30C, the cell retained a high discharge capacity of 54.5 mAh g^–1^, underscoring excellent rate performance and electrochemical stability (Fig. S13). Compared with previously reported gel electrolytes, the Na/CGE/NVP-K_0.05_ cell demonstrated superior cycling stability and high-rate performance, highlighting the significant advantages of CGEs in enhancing the long-term cycling stability and rate capability of solid-state sodium metal batteries [20, 24, 25, 34, 37, 50–65] (Fig. 4e, f). To further evaluate the practical applicability of the composite electrolyte, full cells were assembled with high-mass-loading cathodes for performance evaluation. Notably, under a high mass loading of 7.7 mg cm⁻^2^, the cathode delivered an initial discharge capacity of 104.5 mAh g⁻^1^ at 0.5C (Fig. S14a). Moreover, even at a high rate of 5C, a discharge capacity of 76.6 mAh g⁻^1^ was still maintained (Fig. S14b). This performance is competitive with the state-of-the-art systems reported in the literature, as summarized in Table S3 [20, 66–74]. The influence of electrolyte thickness was examined using a thin film (~ 600 μm, representing a reduction of over 50%). The thinned electrolyte retained excellent performance, achieving a high-capacity retention of 92% after 200 cycles at 5C and delivering a discharge capacity of 68.1 mAh g⁻^1^ even at a high rate of 20C, matching the performance of the ~ 1.35 mm-thick CGE (Fig. S14c, d). These results strongly confirm that the material’s structure forms low-resistance ion “highways,” creating continuous pathways that overcome the limitations of increased diffusion length, further demonstrating its potential for practical applications.

### Disordered and Active Framework Fillers

**Fig. 5:**
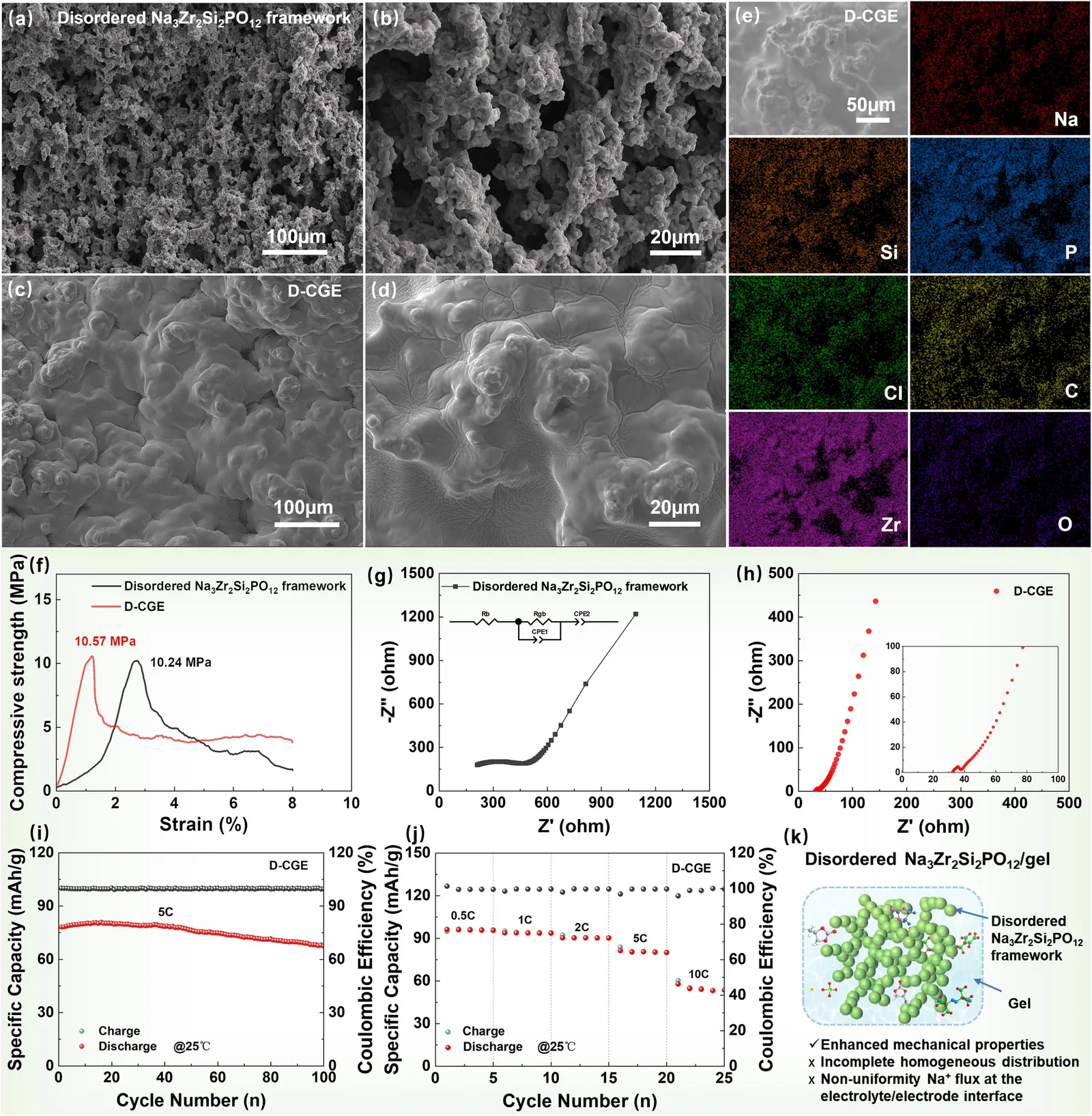
SEM images and EDS mapping: **a**, **b** Disordered Na_3_Zr_2_Si_2_PO_12_ framework, **c**, **d** The fracture surface of D-CGE, **e** EDS mapping of the D-CGE, **f** Compressive strength–strain curves of the disordered Na_3_Zr_2_Si_2_PO_12_ framework and D-CGE, **g** EIS of the disordered Na_3_Zr_2_Si_2_PO_12_ framework at the RT, **h** EIS of the D-CGE at the RT, **i** Cycling performance of the Na/D-CGE/NVP-K_0.05_ cells, **j** Rate capability of the Na/D-CGE/NVP-K_0.05_ full cell from 0.5C to 10C, **k** Schematic illustration of the disordered Na_3_Zr_2_Si_2_PO_12_/gel composite

To elucidate the critical role of structural ordering of the ionic conductor Na_3_Zr_2_Si_2_PO_12_ framework on the electrochemical performance of composite electrolytes, a disordered porous Na_3_Zr_2_Si_2_PO_12_ framework was synthesized as a control sample via a sacrificial template method. SEM images (Fig. 5a, b) reveal a heterogeneous pore size distribution with distinct polydisperse characteristics in the disordered ionic conductor framework. After compounding with the gel electrolyte to form disordered composite gel electrolytes (D-CGE), cross-sectional SEM images (Fig. 5c, d) confirm complete pore filling with no detectable residual porosity. Elemental mapping of C and Cl via EDS further verifies the homogeneous infiltration of the gel phase throughout the porous ceramic matrix (Fig. 5e). To decouple the influence of ceramic content from structural effects, the porosity of the disordered Na_3_Zr_2_Si_2_PO_12_ framework was controlled to match that of the aligned ordered structure, thereby attributing performance differences primarily to the pore architecture. Mechanical testing reveals a fundamental disparity in compressive strength. The ordered Na_3_Zr_2_Si_2_PO_12_ and its composite electrolyte exhibit a strength of 20.11 MPa, substantially higher than the 10.57 MPa of the D-CGE, indicating the inherent mechanical advantage of an aligned structure (Fig. 5f). Furthermore, ion transport properties further highlight this contrast. The intrinsic ionic conductivity of the disordered framework (2.22 × 10⁻^4^ S cm⁻^1^) and that of the D-CGE (2.67 × 10⁻^3^ S cm⁻^1^) are markedly lower than CGE, underscoring the critical importance of directional alignment in establishing continuous and efficient ion-conducting pathways, as shown in Fig. 5g, h. Electrochemical evaluation further underscores the impact of structural ordering. D-CGE retains only 86.2% of its capacity after 100 cycles at 5C, significantly lower than the ordered composite electrolyte (~ 75.9% after 10,000 cycles) (Fig. 5i). Rate capability tests reveal that at a high current density of 10C, D-CGE only delivers a discharge capacity of only 54.6 mAh g⁻^1^, approximately 40% lower than that of the ordered composite gel electrolyte (92.3 mAh g⁻^1^) **(**Fig. 5j**)**. This performance deficit originates from the structural deficiencies of the disordered framework. Although the ceramic phase provides some mechanical reinforcement, its random distribution induces localized stress concentration during cycling. This leads to progressive interfacial contact loss, heterogeneous Na⁺ flux at the electrode–electrolyte interface, accelerated interfacial impedance growth, and ultimately, cell failure, as schematized in Fig. 5k.

### Ion Transport Mechanism

**Fig. 6:**
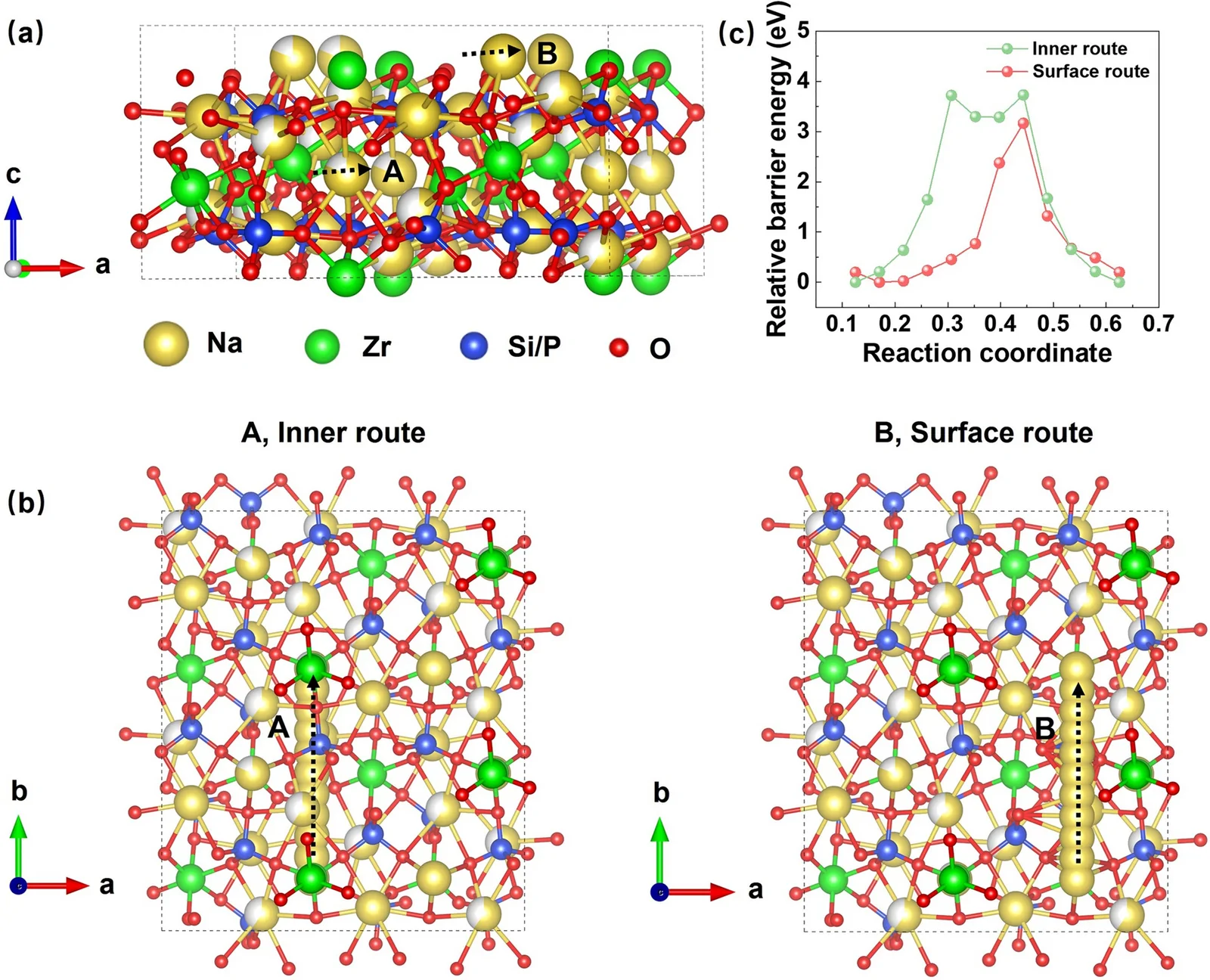
Investigations of the Na-ion conduction mechanism. **a** Crystal structures of Na_3_Zr_2_Si_2_PO_12_ based on first-principles calculations, **b** Na-ion diffusion route in the inner channel (A route) and surface channel (B route), **c** Migration energy barrier in the inner channel and surface channel for Na-ion migration routes, with a step size of 0.05 in fractal coordinates

To investigate the Na-ion transport mechanism in Na_3_Zr_2_Si_2_PO_12_, a 3D ceramic framework was fabricated using freeze-drying, producing a highly exposed crystal surface anticipated to enhance Na^+^ conduction. First-principles density functional theory (DFT) calculations were conducted to examine the Na^+^ migration pathways and their associated energy barriers. Two primary migration routes were identified: route A, which occurs within the ordered bulk channels of the crystal structure, and route B, located at the exposed surfaces or grain boundary regions (Fig. 6a). Structural representations further depict the distinct diffusion pathways, with route A traversing the rigid framework consisting of ZrO_6_ and Si/PO_4_ units, while route B follows less confined, more flexible surface regions (Fig. 6b). Energy barrier profiles indicate that Na^+^ migration along route A faces a higher energy barrier, resulting from spatial restrictions and strong electrostatic interactions within the highly ordered lattice (Fig. 6c) [75, 76]. In contrast, the B route presents a reduced energy barrier, benefiting from local lattice relaxation and topological reconstruction near the surface, which alleviates steric hindrance and creates auxiliary low-barrier pathways [77]. Additionally, undercoordinated atoms at grain boundaries lead to electronic redistribution, further lowering energy peaks and promoting 3D diffusion [64]. The interface between the Na_3_Zr_2_Si_2_PO_12_ surface and the gel phase plays a critical role in enhancing ion transport. Previous studies have indicated that incorporating Na_3_Zr_2_Si_2_PO_12_ fillers into gel electrolytes facilitates the formation of continuous ion conduction pathways at organic–inorganic interfaces [78]. Moreover, interactions between the gel and the ceramic surface establish a synergistic transport environment that further reduces the energy barrier for ion migration compared to single-component systems [79]. Collectively, these findings underscore the significant contribution of both intrinsic surface pathways and extrinsic interfacial effects to the enhancement of ionic conduction. The vertically aligned Na_3_Zr_2_Si_2_PO_12_ framework, fabricated via freeze-drying, not only promotes the prevalence of low-energy surface channels but also provides an ideal scaffold for constructing well-defined interfaces with the gel phase. This hierarchical architecture, integrating the mechanical stability of the ceramic with optimized ion transport along the Na_3_Zr_2_Si_2_PO_12_ surface and across the Na_3_Zr_2_Si_2_PO_12_/gel interface, offers an effective strategy for achieving high ionic conductivity at RT while minimizing interfacial polarization in sodium metal batteries.

### Low-Temperature Electrochemical Performance

**Fig. 7:**
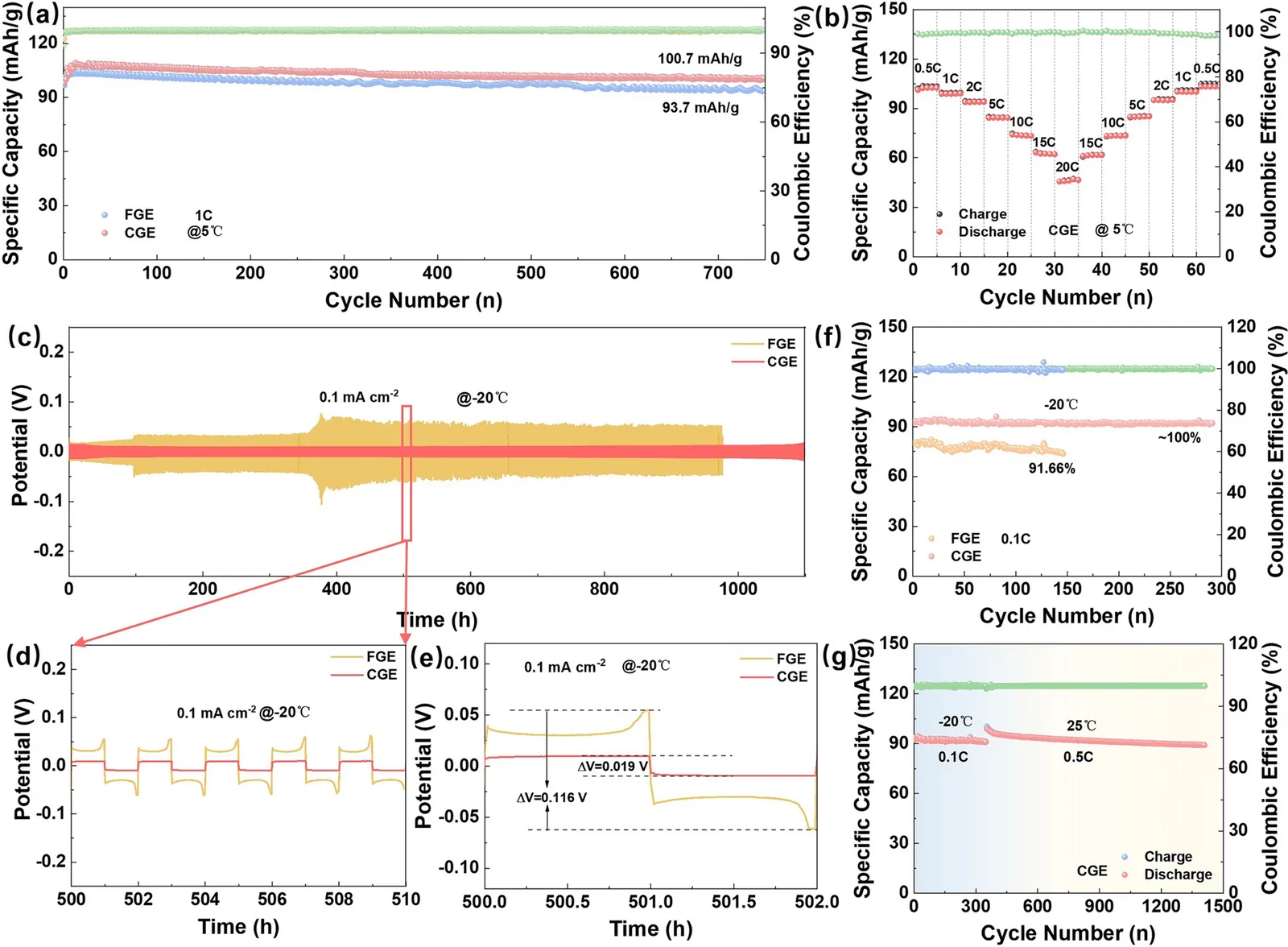
Electrochemical performance tests of FGE and CGE. **a** Comparison of the cycling stability of Na/NVP-K_0.05_ at 1C at 5 °C, **b** Rate performance of the CGE from 0.5C to 20C, **c** Na/Na symmetric batteries tested at 0.1 mA cm^–2^, with magnified views in **d**, **e**; **f** cycling stability of the FGE and CGE at 0.1C at –20 °C, **g** Na/CGE/NVP-K_0.05_ battery initially cycled at –20 °C and subsequently at 25 °C

A systematic study was conducted to comprehensively evaluate the electrochemical performance of CGE in low-temperature environments, with a focus on cycling stability and rate capability in Na/SSEs/Na cells. At 5 °C, the CGE-based cell maintained a specific capacity of 100.7 mAh g^–1^ after 749 cycles, surpassing that of the FGE-based cell, which retained 93.7 mAh g^–1^ (Fig. 7a). Additionally, the rate performance demonstrated that the CGE-based battery sustained a discharge capacity of 45.6 mAh g^–1^ at 20C, attributed to the improved Na-ion transport kinetics facilitated by the SSE (Fig. 7b). To further explore the low-temperature electrochemical behavior of CGE, Na^+^ deposition/stripping characteristics were examined in symmetric cells using both CGE and FGE electrolytes at a current density of 0.1 mA cm^–2^ (Fig. 7c). The FGE system displayed a progressive increase in polarization voltage during cycling, with significant hysteresis voltage fluctuations observed within the first 400 h, eventually stabilizing at about 0.116 V after 500 h (Fig. 7d, e). In contrast, the CGE system exhibited exceptional stability, maintaining a hysteresis voltage as low as 0.019 V after 500 h and remained stable for up to 1,000 h, further confirming the superior performance of the Na/CGE/Na cell. The electrochemical evaluation of the Na/SSEs/NVP-K_0.05_ system also verified its adaptability at low temperatures. Remarkably, the Na/CGE/NVP-K_0.05_ battery maintained a capacity of 99.81 mAh g^–1^ after 291 cycles at –20 °C, with negligible capacity fading. In contrast, the Na/FGE/NVP-K_0.05_ battery showed a capacity retention of only 91.66% after 146 cycles, with the discharge capacity decreasing to 73.6 mAh g^–1^, further emphasizing the superior cycling stability (Fig. 7f). Furthermore, the CGE system displayed excellent rate capability at -20 °C, delivering discharge capacities of 85.5, 72.5, 63.7, 54.0, and 45.7 mAh g⁻^1^ at 0.2C, 0.4C, 0.6C, 0.8C, and 1C, respectively, as shown in Fig. S15. These results collectively provide compelling evidence for the outstanding low-temperature performance of the composite electrolyte. To evaluate the electrochemical stability of CGE under rapid temperature fluctuations, a temperature shock test was performed. Following 350 cycles at –20 °C, the battery was immediately subjected to cycling at 0.5C at 25 °C (Fig. 7g). The battery retained a discharge capacity of 90.8 mAh g^–1^ after 350 cycles at –20 °C and maintained stability with a capacity of 89.1 mAh g^–1^ over 1,000 cycles at 25 °C. The stable capacity retention of the Na_3_Zr_2_Si_2_PO_12_/Gel composite at low temperatures originates from multilevel, synergistic effects. This hierarchical design first ensures efficient ion transport: the vertically aligned Na_3_Zr_2_Si_2_PO_12_ framework provides a rigid “ionic highway” resilient to temperature fluctuations, securing continuous conduction, while the gel phase infiltrating its pores further enhances kinetics via low-activation-energy pathways [80, 81]. In addition, the soft gel accommodates volumetric fluctuations between electrodes and electrolyte, ensuring durable contact and substantially reducing interfacial impedance [85, 86]. This unique combination of rigid and soft components ultimately yields exceptional structural and thermal integrity against low-temperature stresses, with the synergy between facilitated ion transport, stabilized interfaces, and mechanical robustness collectively enabling high performance. This robust performance highlights the structural integrity and excellent reversibility of the CGE system under abrupt environmental changes, further demonstrating its suitability for practical applications in extreme conditions.

### Dendrite Suppression and Interface Stabilization Mechanisms

**Fig. 8:**
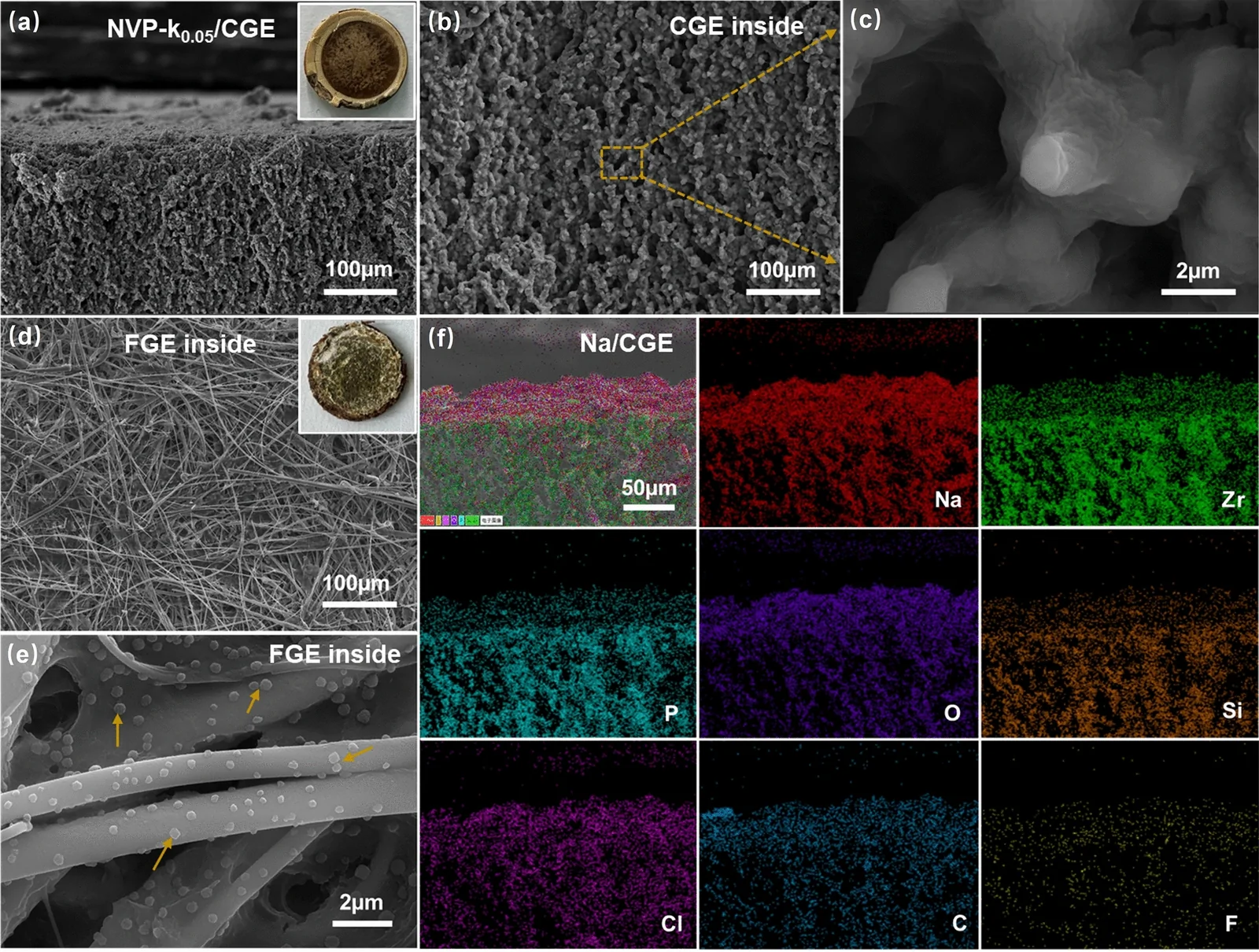
SEM images of the Na/NVP-K_0.05_ full cell after cycling at 60 °C. **a** NVP-K_0.05_/CGE cross section, **b**, **c** Cross section of the CGE, **d**, **e** FGE cross section, **f** EDS mapping of the Na/CGE cross section

To further elucidate the intrinsic mechanisms underlying the stability of the composite gel electrolytes, a systematic SEM analysis was performed on Na/CGE/NVP-K_0.05_ and Na/FGE/NVP-K_0.05_ cells after prolonged cycling at 60 °C. SEM images revealed that cross-sectional CGE/NVP-K_0.05_ retained excellent structural integrity even after extended cycling, with an inset showing a photograph of the electrolyte after cycling (Fig. 8a). As shown in Fig. 8b, c, the cross-sectional analysis of the CGE electrolyte showing an absence of dendrite intrusion as evidenced by imaging. This finding is further corroborated by EDS analysis performed at both the anode/electrolyte and cathode/electrolyte interfaces (Figs. 8f and S16). These results confirm that the composite electrolyte effectively suppresses dendrite formation during long-term cycling, thereby significantly minimizing the risk of uncontrolled sodium dendritic propagation through the electrolyte structure. However, SEM analysis revealed the presence of particles of diverse sizes distributed throughout the interior of the FGE, encompassing both fiber and gel surfaces, with an inset showing a photo of the electrolyte after cycling (Fig. 8d, e). As confirmed by EDS analysis (Fig. S17), these particulate features are predominantly composed of metallic sodium. Specifically, EDS point scan tests were performed on the particles deposited on the glass fiber surface, and the results indicate that the atomic ratios of C, O, and Na are 37.11%, 37.72%, and 17.32%, respectively (Fig. S17c). Notably, only a trace amount of Na originates from NaClO_4_ in the gel, while the vast majority of Na is attributed to sodium deposition products. The relatively high atomic ratios of C and O are likely derived from Na_2_O and Na_2_CO_3_, which form via the oxidation of deposited metallic sodium in air during sample transfer and characterization processes. To further understand the role of the composite structure in regulating deposition morphology, the cycled CGE-Na interface after 2000 cycles at 0.1 mA cm⁻^2^ was examined by SEM (Fig. S18). The results reveal that sodium deposits in the CGE system exhibit a flat, planar morphology tightly adhered to the electrolyte surface, forming a uniform deposition layer. Furthermore, X-ray photoelectron spectroscopy (XPS) was employed to analyze the chemical composition of the cycled sodium metal surface (Fig. S19). Compared to pristine Na, the CGE-Na surface shows distinct chemical characteristics: the Na 1 s spectrum displays a NaF characteristic peak; the O 1 s spectrum exhibits three peaks corresponding to Na₂O (530.7 eV), C–O (532.4 eV), and the Na Auger peak (535.2 eV); the C 1* s* spectrum shows characteristic peaks at 284.1, 285.5, 287.7, and 289.1 eV, assigned to C–C, C–O, C = O, and O–C = O species, respectively. These chemical species originate from the reductive decomposition of electrolyte components including ethylene carbonate (EC), propylene carbonate (PC), and fluoroethylene carbonate (FEC). These findings demonstrate that the CGE effectively modulates the composition of the solid electrolyte interphase (SEI) on the sodium metal surface by introducing fluorine-containing inorganic phases (NaF) and reconstructing an organic–inorganic hybrid SEI structure. The organic components provide excellent flexibility and interfacial compatibility, buffering volume changes during sodium plating/stripping, while the fluorine-containing inorganic phases enhance interfacial ion transport properties, synergistically achieving stabilization of the electrode–electrolyte interface. Conventional commercial separators are typically non-ionic conductors and suffer from insufficient mechanical strength. To overcome these limitations, a highly conductive Na₃Zr₂Si₂PO₁₂ framework was incorporated into the composite gel electrolyte, simultaneously constructing a continuous ionic conduction network and markedly enhancing its mechanical robustness. This network maintains structural integrity during cycling, thereby effectively suppressing abnormal sodium deposition and dendrite growth. Furthermore, compared with dense ceramics, the composite gel electrolyte not only combines the flame-retardant properties of ceramics with the high ionic conductivity of ionic liquids but also achieves an approximate 20% reduction in mass, further improving the energy density of the battery (Fig. S20 and Table S4). This study establishes compelling economic advantages through innovations in materials selection and processing techniques. The scalable manufacturing approach employs low-cost commercial raw materials, eliminating the requirement for expensive polymer binders and inert substrates typically used in conventional slurry-cast methods. Concurrently, the optimized sintering protocol enhances production efficiency substantially while maintaining structural integrity. These achievements provide crucial experimental evidence and innovative design principles for developing safe, long-life sodium metal batteries, while also enhancing lifecycle cost-effectiveness.

## Conclusion

In conclusion, this study provides a systematic evaluation of the composite gel electrolyte. DFT calculations reveal that the vertically aligned Na₃Zr₂Si₂PO₁₂ framework, which features extensively exposed crystal surfaces, offers low-energy barrier pathways and establishes a graded diffusion network, thereby effectively facilitating Na⁺ transport. Benefiting from this structural design, the CGE enables efficient stress transfer, achieving a compressive strength of 20.1 MPa, while maintaining excellent ionic conductivity and effectively suppressing sodium dendrites. Furthermore, the 3D-Na₃Zr₂Si₂PO₁₂ framework further serves as a thermal barrier, imparting the CGE with superior flame retardancy. This work confirms the structural integrity of the CGE and its ability to facilitate sodium deposition, further substantiating its effectiveness in suppressing dendrites growth. Moreover, Na/CGE/NVP-K_0.05_ cells exhibit 75.9% capacity retention after 10,000 cycles at 5C (25 °C) and deliver 78.5 mAh g^−1^ even at 30C (60 °C). Notably, the CGE exhibits excellent low-temperature adaptability, retaining nearly 100% capacity at –20 °C. These results highlight the CGE as a highly promising candidate for next-generation high-performance sodium metal batteries, offering significant advancements in safety, stability, and environmental adaptability for energy storage applications.

## Supplementary Information

Below is the link to the electronic supplementary material.

Supplementary file1 (DOCX 7164 KB)

Supplementary file2 (MP4 3381 KB)

Supplementary file3 (MP4 1888 KB)

Supplementary file4 (MP4 2331 KB)
