# Supplementary material for: High-Strength 3D-Ordered Ceramic-Gel Composite Electrolytes Enable Highly Stable Sodium Metal Batteries at − 20 to 60 °C
Source: Nanomicro Lett. 2026 Jan 4;18:195. doi: 10.1007/s40820-025-02032-4 (PMC12765760; doi:10.1007/s40820-025-02032-4)
Supplement: Supplementary file 1 — Supplementary file1 (DOCX 7164 KB) [file 40820_2025_2032_MOESM1_ESM.docx]

Supporting Information for

**High-Strength 3D-Ordered Ceramic-Gel Composite Electrolytes Enable Highly Stable Sodium Metal Batteries at −20 to 60 °C**

Liying Shen^1,2,4^, Chuyan Hu^1^, Zhenhui Huang^1^, Jiarui Yang^1^, Yanwei Jia^1^, Yufeng Zhao^3^*, Rüdiger Berger^2^, Qiang Liu^1,4^*, Yu Zhou^1,4^

^1^State Key Laboratory of Precision Welding & Joining of Materials and Structures, School of Materials Science and Engineering, Harbin Institute of Technology, Harbin 150001, P. R. China

^2^Max Planck Institute for Polymer Research, Mainz, 55122, Germany

^3^College of Sciences and Institute for Sustainable Energy, Shanghai University, Shanghai 200444, P. R. China

^4^Institute for Advanced Ceramics, Key Laboratory of Advanced Structural-Functional Integration Materials & Green Manufacturing Technology, School of Materials Science and Engineering, Harbin Institute of Technology, Harbin 150001, P. R. China

*Corresponding authors. E-mail: [qiangliu@hit.edu.cn](mailto:qiangliu@hit.edu.cn) (Qiang Liu); [yufengzhao@shu.edu.cn](mailto:yufengzhao@shu.edu.cn) (Yufeng Zhao)

**Supplementary Figures and Tables**


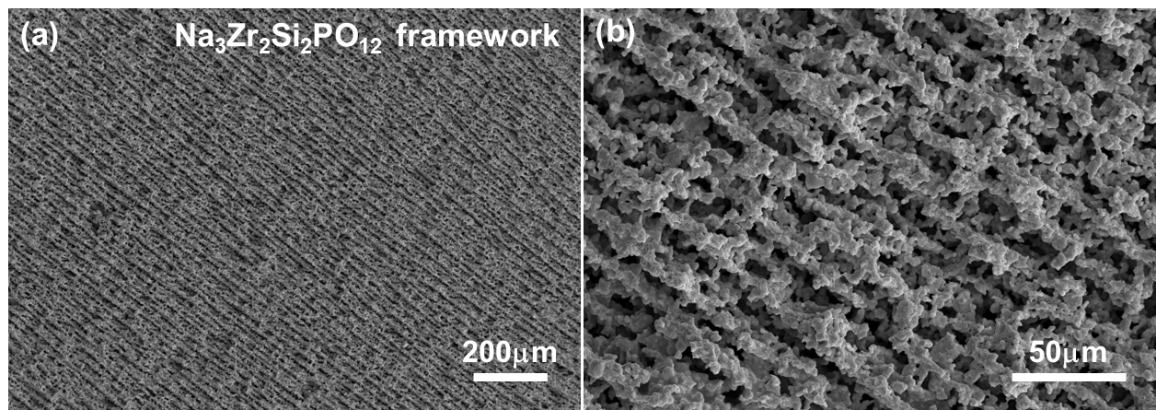


**Fig. S1** **a, b** SEM images of the Na₃Zr₂Si₂PO₁₂ framework acquired at a 45° viewing angle to reveal its 3D morphology (the described "45° tilt" was a deliberate adjustment of the SEM sample stage, not a measurement of the material's intrinsic angle)


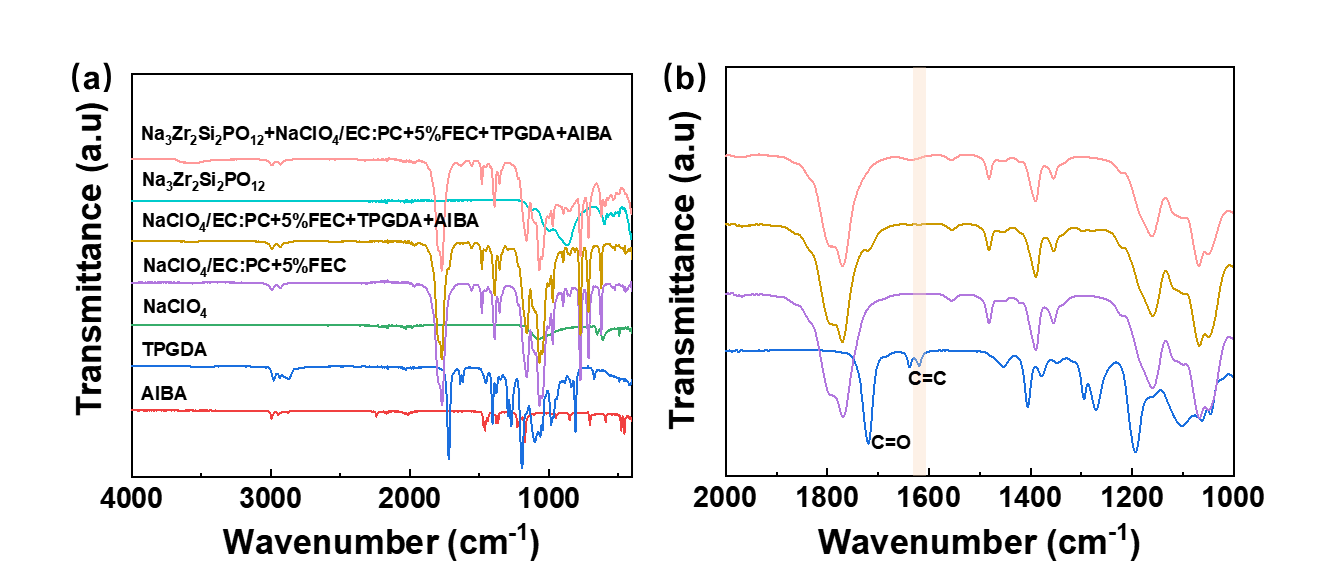


**Fig. S2** **a** FTIR spectra and a magnified area of **b**


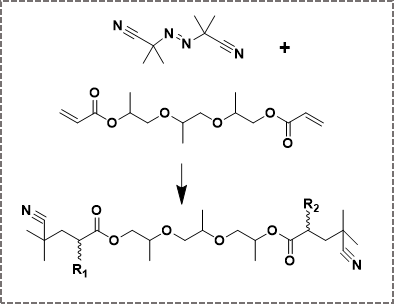


**Fig. S3** The polymerization of the TPGDA monomers initiated by AIBN


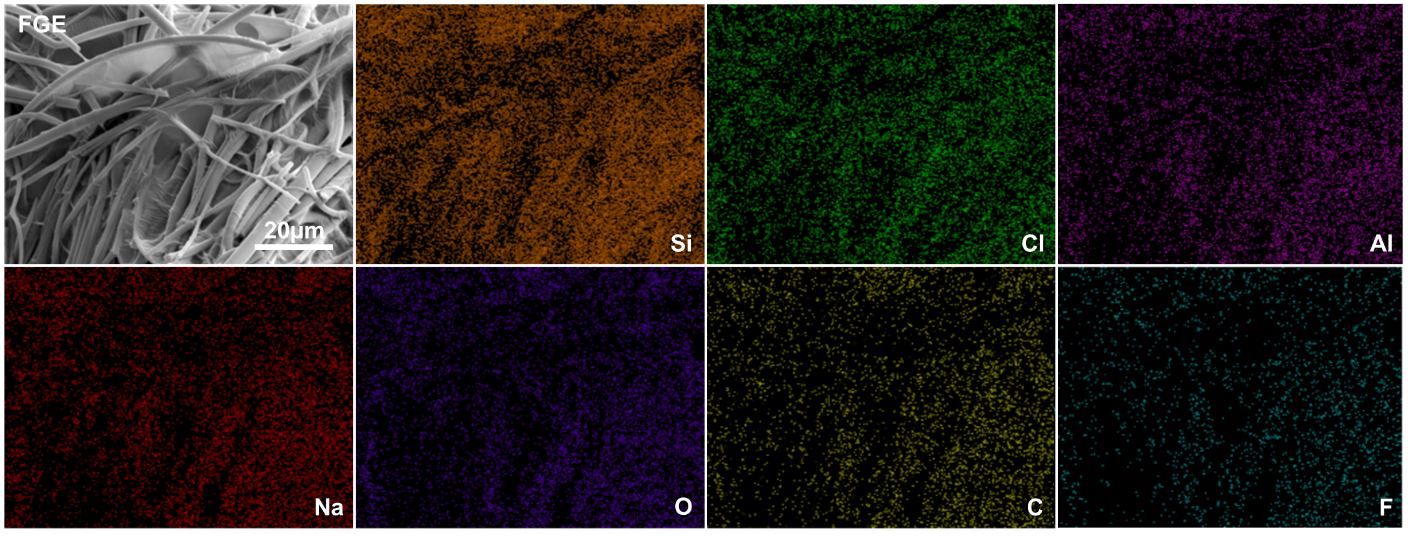


**Fig. S4** EDS mapping of the FGE


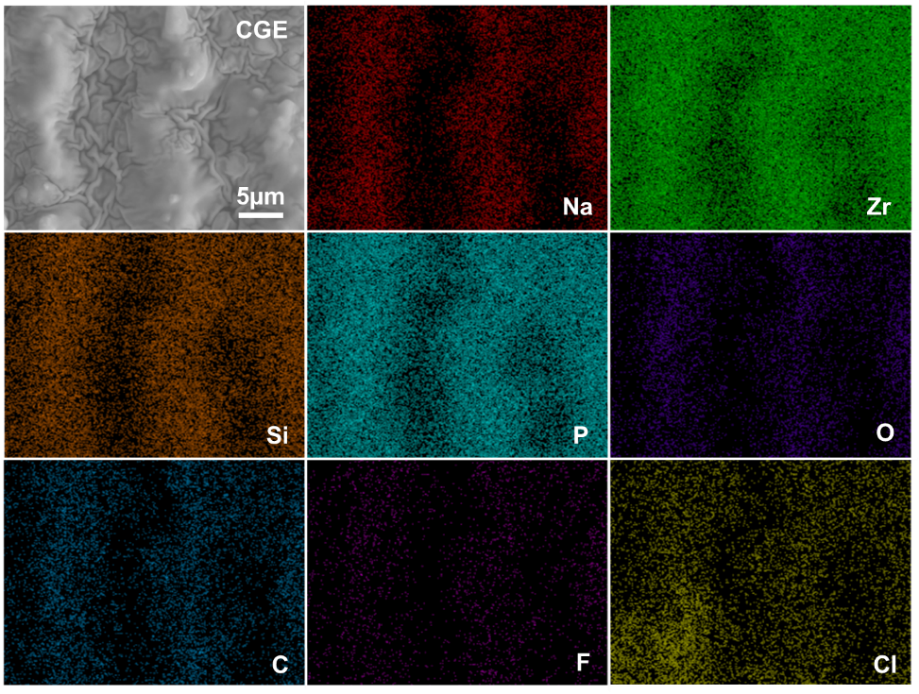


**Fig. S5** EDS mapping of the CGE


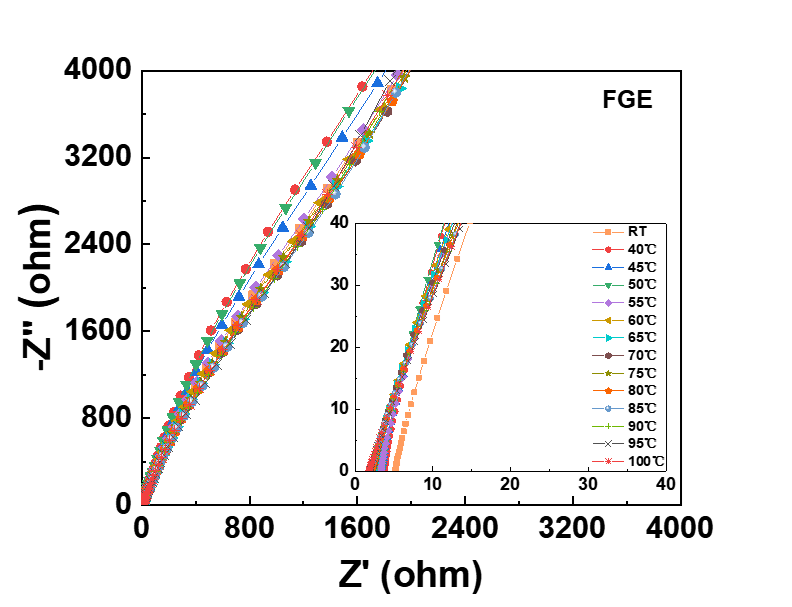


**Fig. S6** EIS of FGE at different temperatures with a thickness of 0.1 mm and a diameter of 16 mm


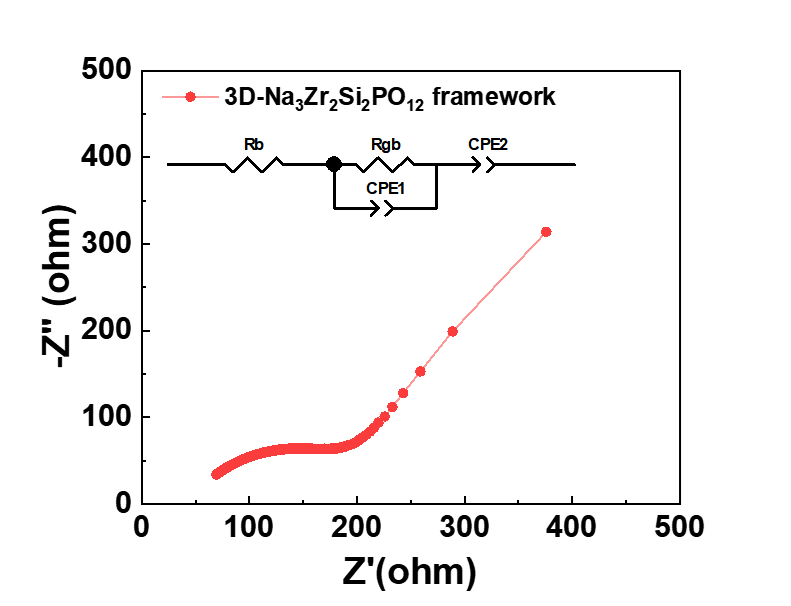


**Fig. S7** EIS of the 3D-Na₃Zr₂Si₂PO₁₂ framework at the RT


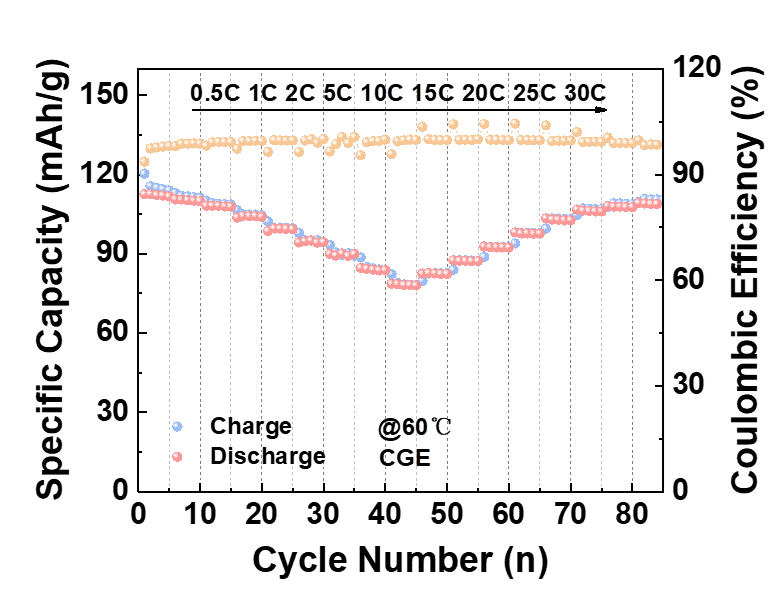


**Fig. S8** Rate performance of Na/CGE/NVP-K_0.05_ at 60 ℃


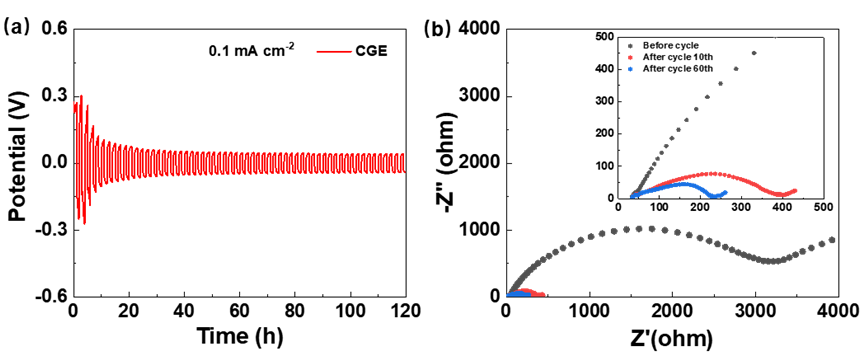


**Fig. S9** Interfacial stability of the symmetric Na/Na cell with CGE. **a** Na/Na symmetric battery tested at 0.1 mA cm^–2^. **b** Corresponding EIS spectra measured at different cycle numbers at RT
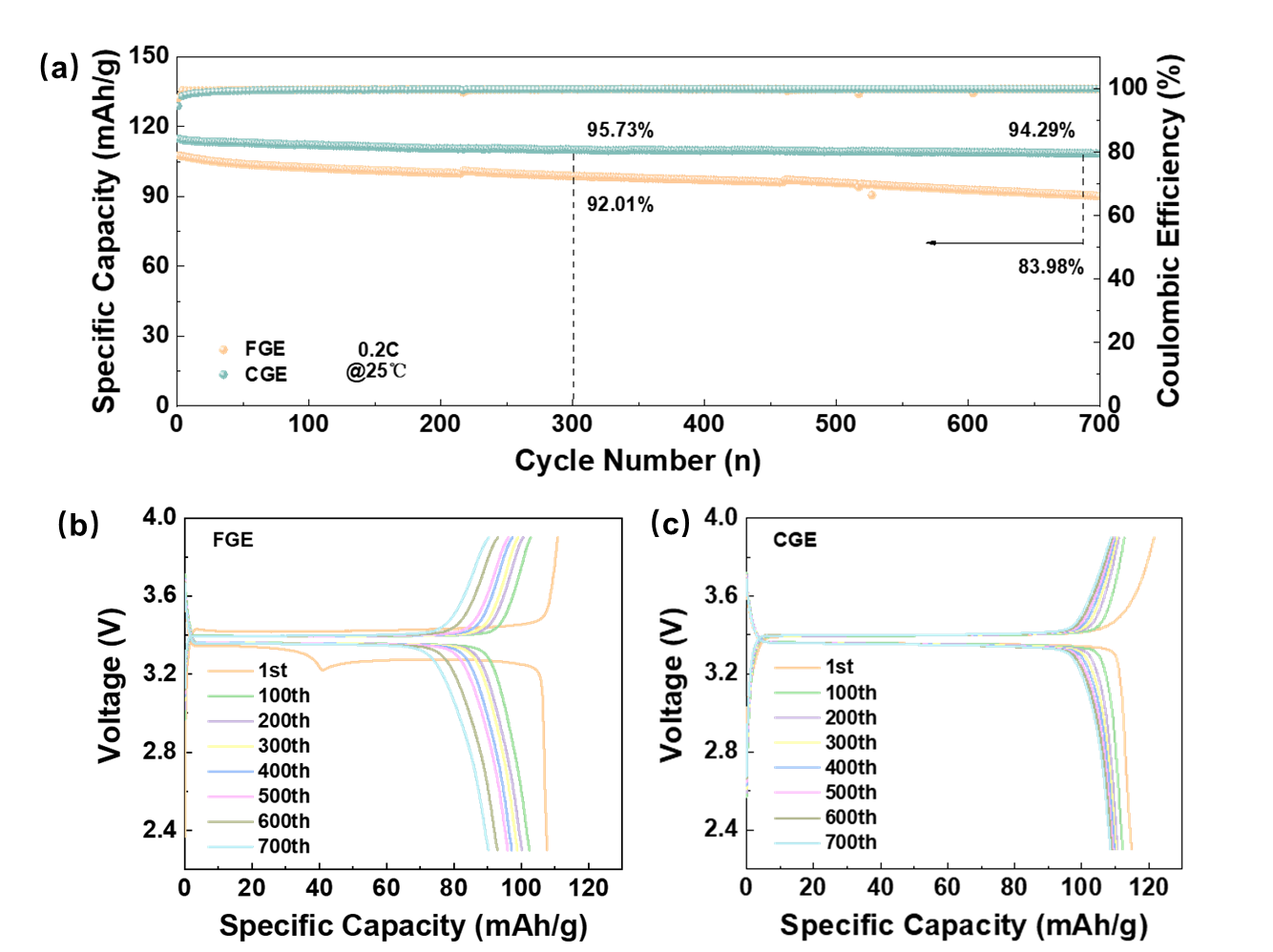


**Fig. S10** **a** Cycling performance of Na/FGE/NVP-K_0.05_ and Na/CGE/NVP-K_0.05_ full battery at 0.2C under 25 ℃ and charge/discharge profiles **b, c**


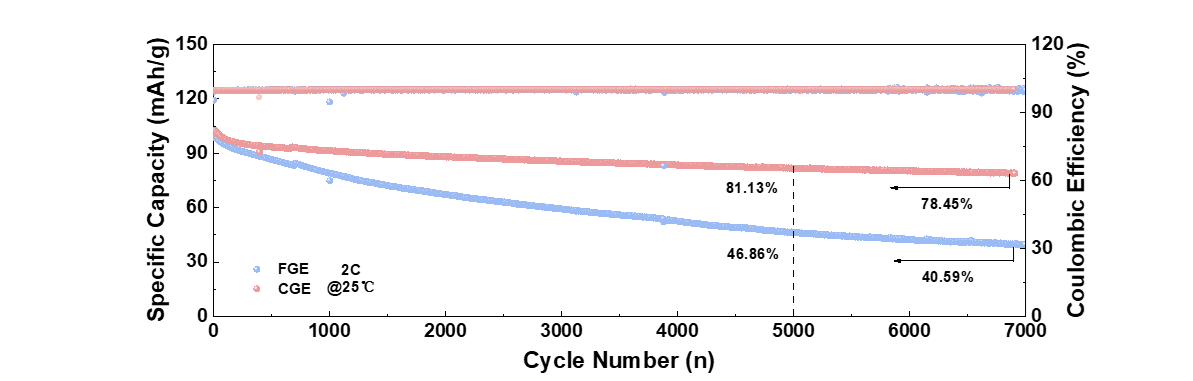


**Fig. S11** Long-term cycling of Na/FGE/NVP-K_0.05_ and Na/CGE/NVP-K_0.05_ full battery at 25 ℃

**Fig. S12** The charge/discharge voltage profiles at different cycles of the Na/FGE/NVP-K_0.05_ full battery


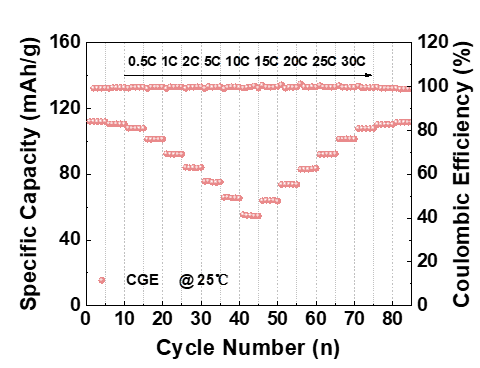


**Fig. S13** Rate performance of Na/CGE/NVP-K_0.05_ full battery at 25 ℃


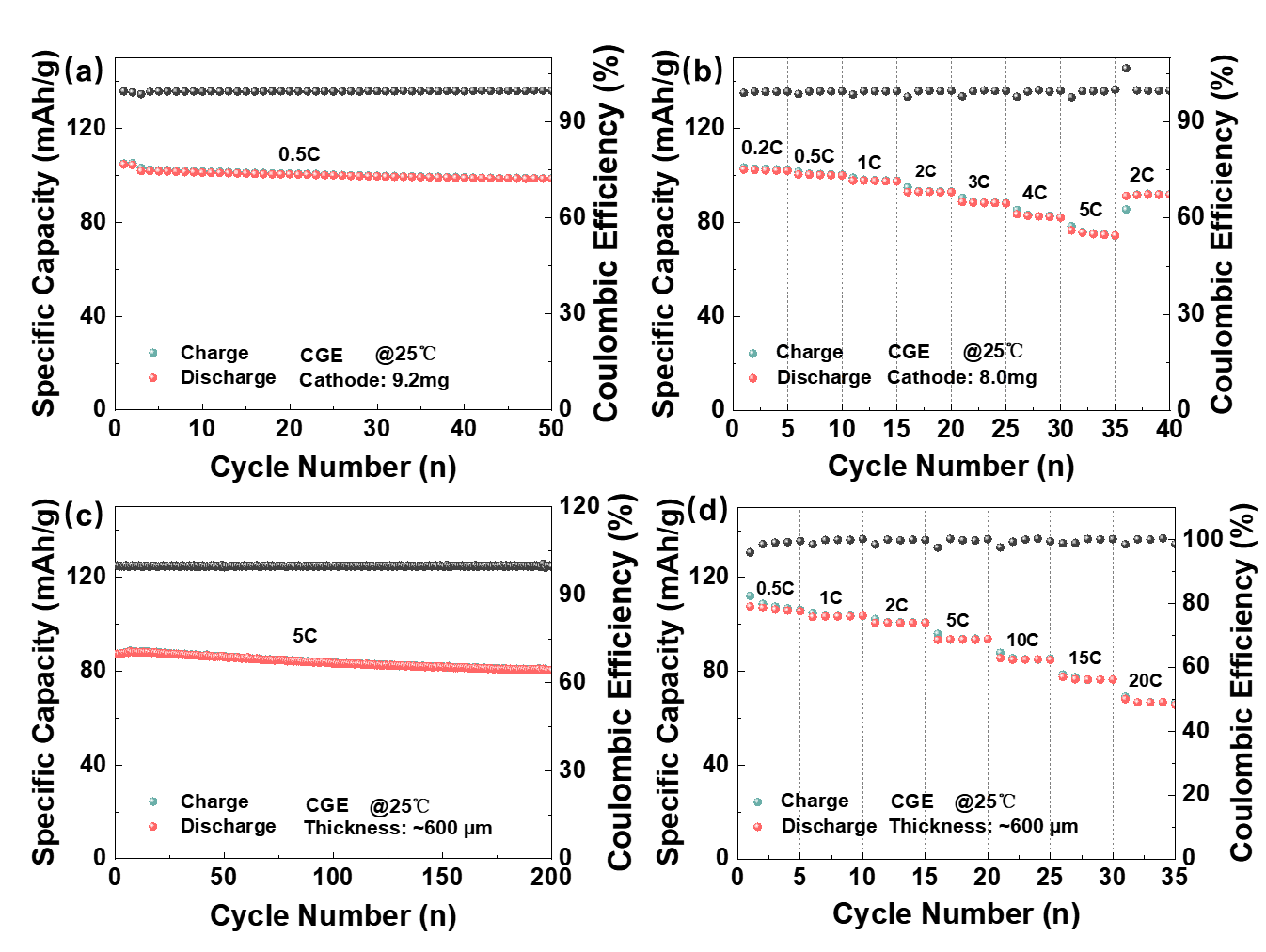


**Fig. S14** Electrochemical performance of the Na/CGE/NVP-K₀.₀₅ full cell at 25 °C. **a** Cycling performance and **b** rate capability of a cell using a high-mass-loading cathode material. **c** Cycling performance and **d** rate capability of a cell assembled with a thin CGE electrolyte pellet (~600 μm)


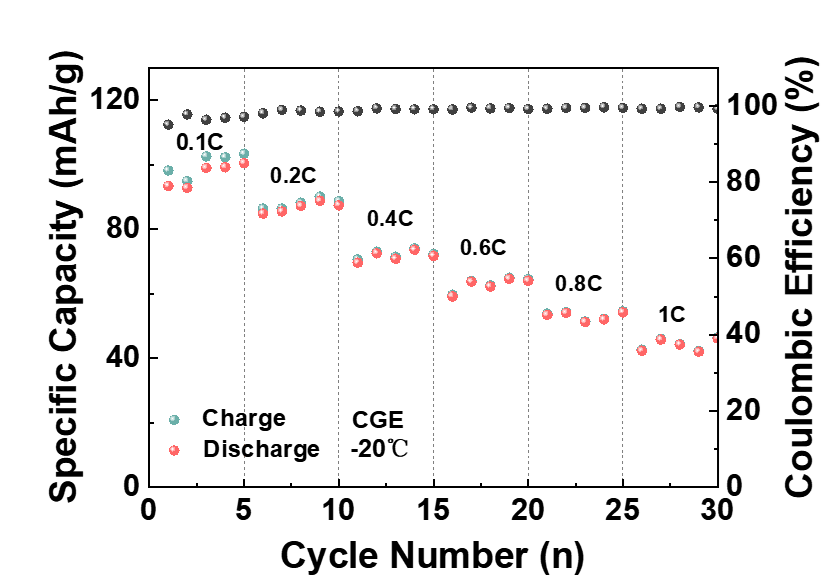


**Fig. S15** Rate performance of the CGE at -20 °C


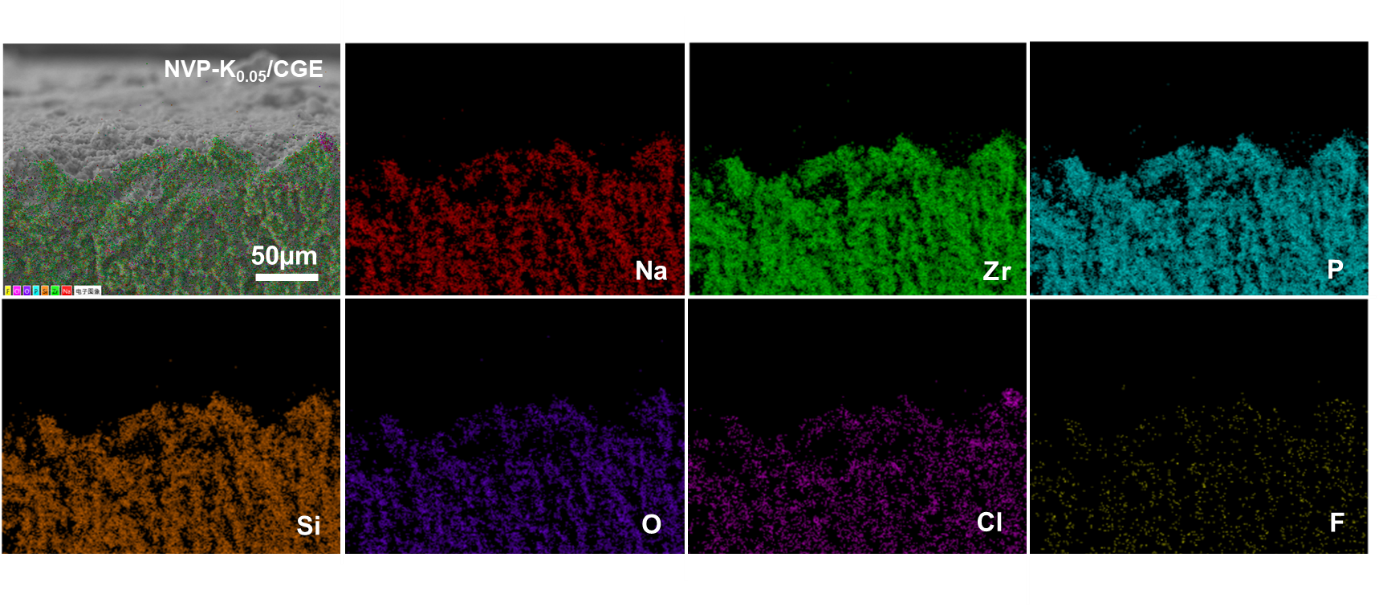


**Fig. S16** EDS mapping of the CGE cross-section of the Na/CGE/NVP-K_0.05_ full battery after cycles at 60 °C


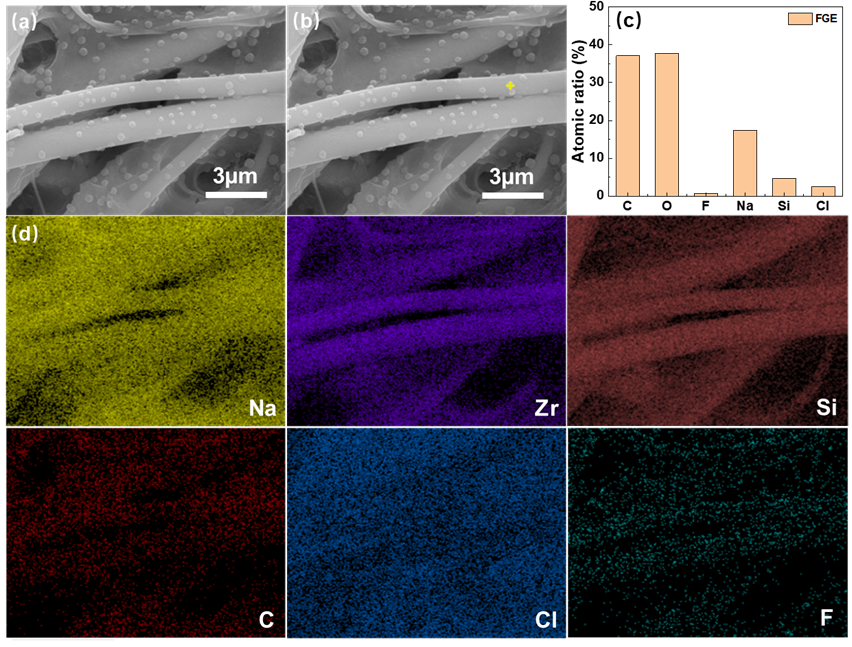


**Fig. S17** SEM images and EDS mapping of the FGE cross-section of the Na/FGE/NVP-K_0.05_ battery cell after cycles at 60 °C. **a,b** SEM images and **c,d** EDS elemental analysis


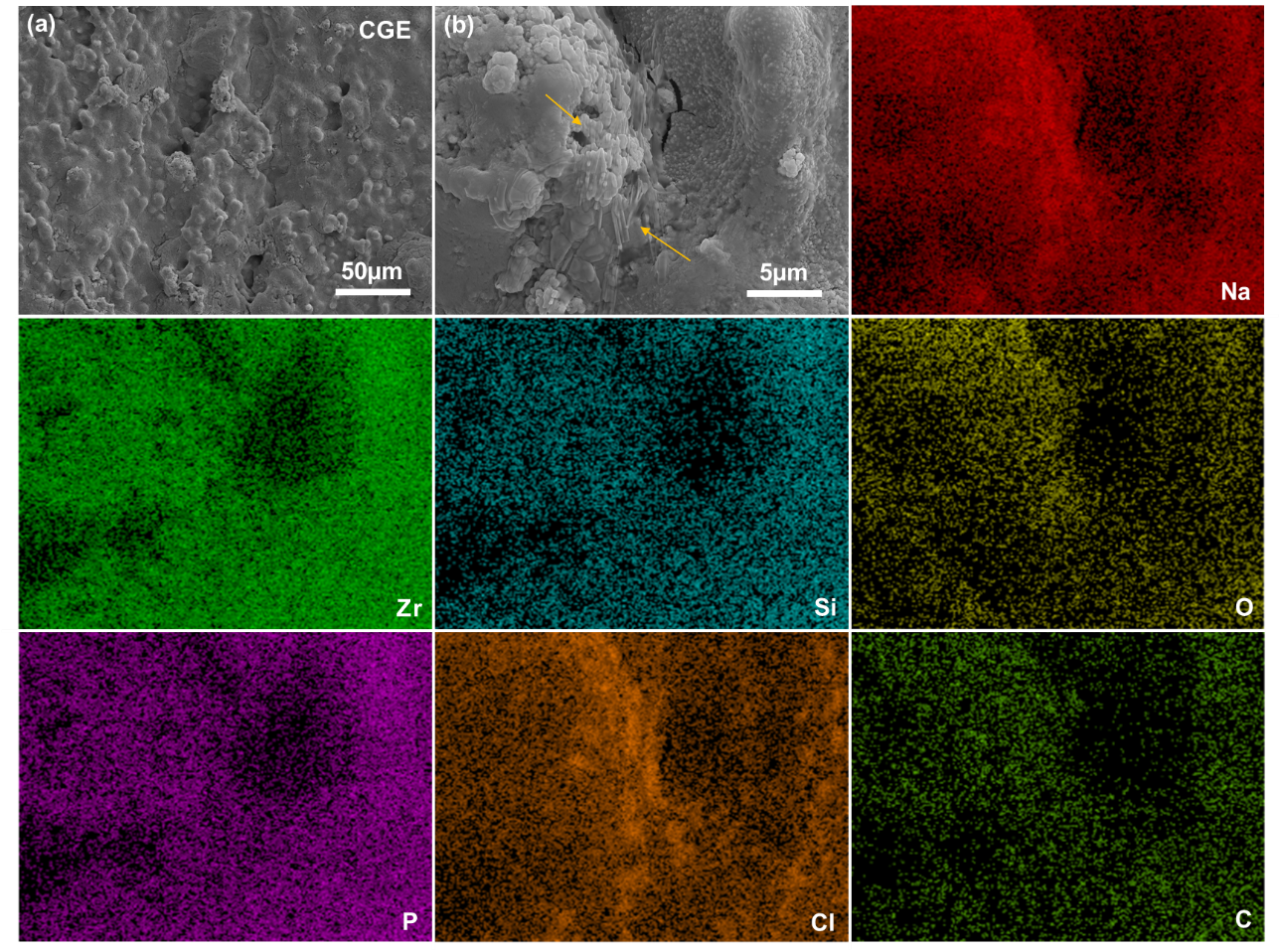


**Fig. S18**Morphological and elemental analysis after cycling in a symmetric Na/CGE/Na cell. **a** Top-view SEM image. **b** Corresponding EDS elemental mapping


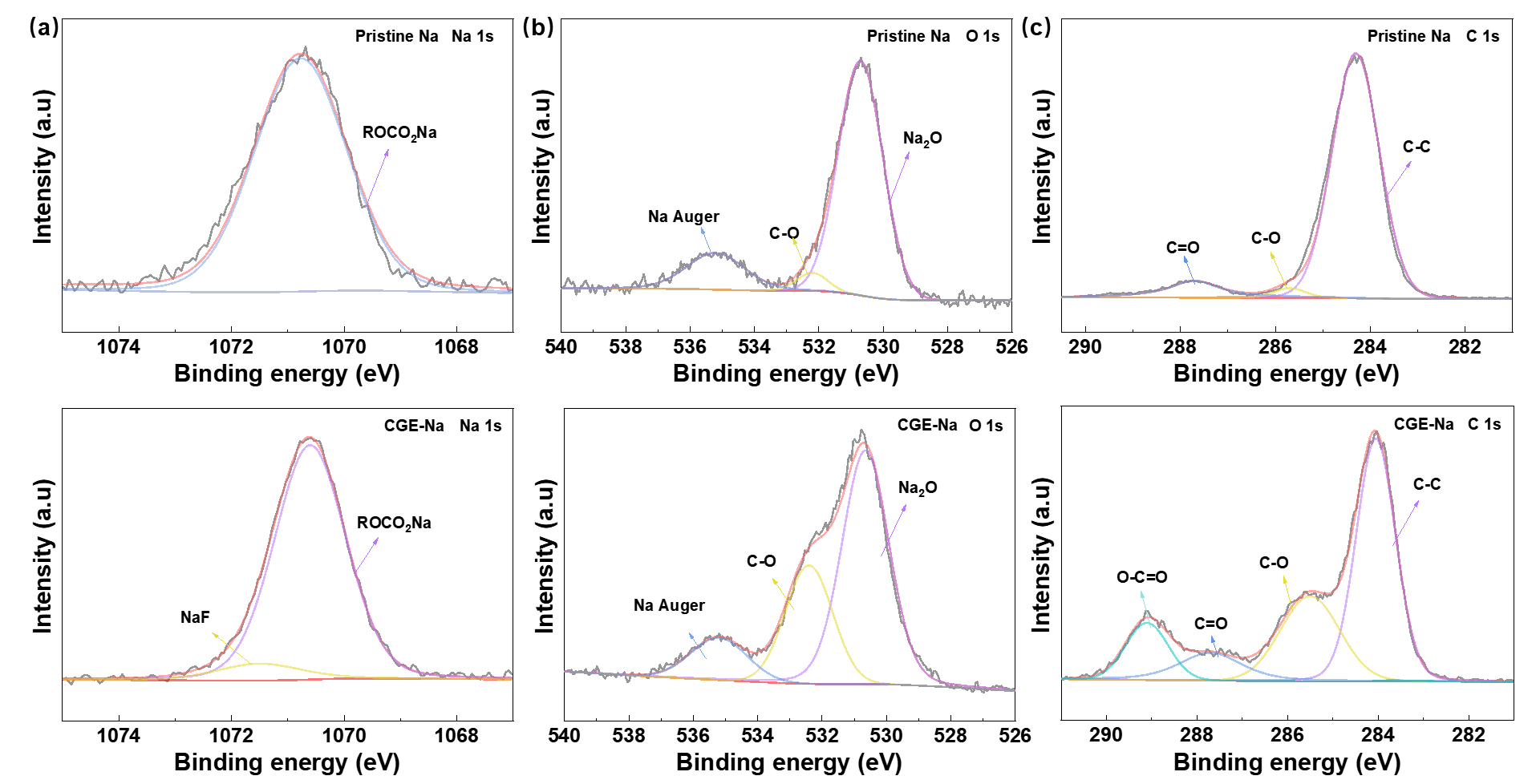


**Fig. S19**XPS of pristine Na and cycled Na electrode in Na/CGE/Na cell. **a** Na 1s. **b** O 1s. **c** C1s


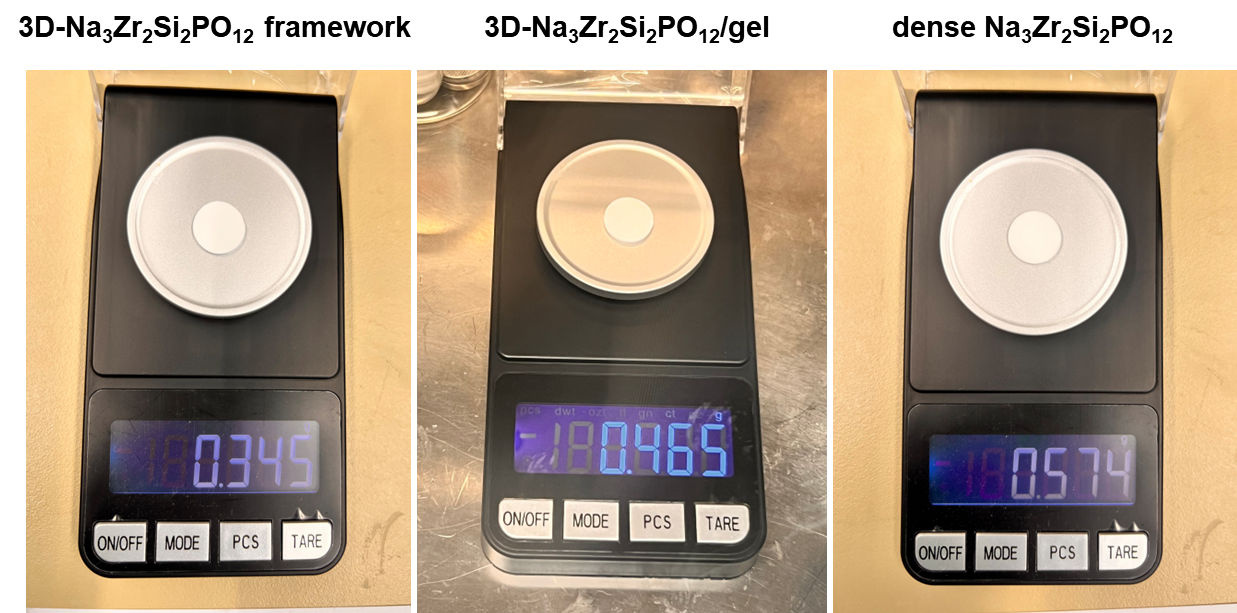


**Fig. S20** Mass comparison of the 3D-Na₃Zr₂Si₂PO₁₂ framework, 3D-Na₃Zr₂Si₂PO₁₂/gel and dense Na₃Zr₂Si₂PO₁₂ ceramic under the same thickness and diameter

**Table S1** Ionic conductivity and corresponding parameters of the 3D-Na₃Zr₂Si₂PO₁₂ framework

| **Sample** | **L/mm** | **R/mm** | **R/Ω** | **σ/(S cm^-1^)** |
| --- | --- | --- | --- | --- |
| 3D-Na₃Zr₂Si₂PO₁₂ framework | 1.25 | 1.51 | 211 | 3.31 x 10^-4^ |

**Table S2** Mechanical properties and ionic conductivity of reported composite gel electrolytes

| **Materials** | **Mechanical strength** | **Ionic conductivity**  **(S cm⁻¹)** | **Refs.** |
| --- | --- | --- | --- |
| PPC37 (GPEs) | 2.43 MPa (Fracture strength) | 1.06 x 10⁻^3^ (25 ℃) | [S1] |
| PGT32-5% | 0.55 MPa (tensile strength) | 9.1 x 10⁻^4^ (27 ℃) | [S2] |
| IL-PVdF-HFP | 0.45 MPa (tensile strength) | 4.0 x 10⁻^4^ (25 ℃) | [S3] |
| U-HCGPE | 9.9 MPa (tensile strength) | 7.89 x 10⁻^4^ (RT) | [S4] |
| PVA-PAA-LiCl-KOH | 1.34 MPa (tensile strength) | 3.18 x 10⁻^3^ (RT) | [S5] |
| SCGPE | 20.5 MPa (Fracture strength) | 1.73 x 10⁻^3^ (30 ℃) | [S6] |
| Glass fiber (GF) | 2 MPa | 2.6 x 10⁻^3^ (25 ℃) | [S7] |
| Plasticized crosslinked PEO membranes (PEO_m_) | ~1 MPa (tensile strength) | 2.0 x 10⁻^4^ (20 ℃) | [S8] |
| NW/P(VDF-HFP) | 29 MPa (Fracture strength) | 8.2 x 10⁻^4^ (RT) | [S9] |
| PEO/NBR | 0.69 MPa (tensile strength) | 2.4 x 10⁻^3^ (RT) |  |
| NBR | 0.87 MPa (tensile strength) | 4.0 x 10⁻^4^ (RT) | [S10] |
| PEO | 0.24 MPa (tensile strength) | 3.1 x 10⁻^3^ (RT) |  |
| CGE | 20.11 MPa  (Compressive strength) | 3.37 x 10⁻^3^ (RT) | This work |
| 3D/Na₃Zr₂Si₂PO₁₂ framework | 19.18 MPa  (Compressive strength) |  | This work |

**Table S3** Comparison of the electrochemical performance of the NVP-K_0.05_/CGE/Na sodium metal battery and reported solid-state (or quasi-solid) batteries with high-mass-loading cathodes

| **Cell** | **Electrolyte** | **Performance** | **Mass-loading** | **Temp.** | **Refs.** |
| --- | --- | --- | --- | --- | --- |
| NMT/Na | GPE | ~83 mAh g^-1^ (1 C) | 9.07 mg cm^-2^ | RT | [S11] |
| NVP/Na | QSE-T | ~85 mAh g^-1^ (0.2 C) | 8.2 mg cm^-2^ | RT | [S12] |
| NVP/Na | PVDF GPE | 75.8 mAh g^-1^ (0.5 C) | 3.01 mg cm^–2^ | 23 °C | [S13] |
| NVP/Na | CPE | ~95 mAh g^-1^ (3 C) | 10.0 mg cm^-2^ | 25 °C | [S14] |
| NNM/Na | PEO-NZSP | 43.2 mAh g^-1^ (3 C) | 3-4 mg cm^-2^ | 55 °C | [S15] |
| NVP/Na | PDA@PU-GPE | 86.46 mAh g^-1^ (1 C) | ∼8.1 mg cm^-2^ | RT | [S16] |
| NVP/Na | QSE | 103 mAh g^-1^ (0.2 C) | 10.9 mg cm^-2^ | RT | [S17] |
| NVP/Na | PLA-NaF GPE | 107.3 mAh g^-1^ (0.5 C) | 3.3 mg cm^-2^ | 25 °C | [S18] |
| NVP@C/Na | HSE | 98 mAh g^-1^ (0.2 C) | ~3.0 mg cm^–2^ | RT | [S19] |
| NVP/Na | PSSIA-NZSP | 94.6 mAh g^-1^ (0.5 C) | 5.6 mg cm^-2^ | 25 °C | [S20] |
| NVPK-_0.05_/Na | CGE | 104.5 mAh g⁻¹ (0.5 C) | 7.7 mg cm⁻² | 25 °C | This work |
|  |  | 76.6 mAh g⁻¹ (5 C) | 6.7 mg cm⁻² |  |  |

**Table S4** Comparison of weight reduction between the 3D-Na₃Zr₂Si₂PO₁₂ framework and dense Na₃Zr₂Si₂PO₁₂ ceramic

| **Samples** | **3D****-Na_3_Zr_2_Si_2_PO_12_ framework** | **3D-Na_3_Zr_2_Si_2_PO_12_/gel** | **Dense Na₃Zr₂Si₂PO₁₂** |
| --- | --- | --- | --- |
| R/mm | 14 | 14 | 14 |
| H/mm | 1.28 | 1.28 | 1.28 |
| m/g | 0.354 | 0.465 | 0.574 |
| Porosity/% | ~47% | / | ~7% |

All pellets were fabricated with identical geometry (diameter 14 mm, thickness 1.28 mm). The sample volume was calculated according to the following equation (S1):

$$V=\pi\left( \frac{D}{2} \right)^{2}H (S1)$$

Where 𝐷 is the pellet diameter (cm) and 𝐻 is the thickness (cm).

The bulk density of each sample was determined by the following equation (2):

$$\rho=\frac{m}{V} (S2)$$

Where 𝑚 is the measured mass (g), and V is the volume of the sample (cm³).

The porosity (𝜙) of the 3D-Na₃Zr₂Si₂PO₁₂/gel was determined using the bulk density and the theoretical density of Na₃Zr₂Si₂PO₁₂, according to Equation (3):

$$\phi=1-\frac{\rho_{b}}{\rho_{T}} (S3)$$

where *ϕ* is the porosity (%), $\rho_{b}$ is the bulk density (g·cm⁻³) determined from the sample mass and geometric volume, and $\rho_{T}$ ​ is the theoretical density (g·cm⁻³), which was obtained from crystallographic data or estimated based on the density of dense ceramics.

The mass-reduction percentage of the composite gel electrolyte (CGE) relative to the dense ceramic was calculated using Equation (4):

$$Mass reduction (\%)=\frac{m_{d}-m_{CGE}}{m_{d}}\times100\% (S4)$$

where $m_{d}$ is the mass of dense Na₃Zr₂Si₂PO₁₂ ceramic (g), $m_{CGE}$ is the mass of 3D-Na₃Zr₂Si₂PO₁₂/gel composite gel electrolyte.

The 3D-Na₃Zr₂Si₂PO₁₂ framework was prepared by a freeze-drying method, while the dense ceramic was synthesized via the solid-state reaction method. To ensure identical pellet dimensions for comparison, both the porous framework and the dense ceramic were processed using a wire-cutting technique. The weight analysis for both the 3D-Na₃Zr₂Si₂PO₁₂ framework and the 3D-Na₃Zr₂Si₂PO₁₂/gel composite electrolyte was carried out on the identical physical sample to guarantee accuracy. Specifically, the mass was measured for the bare framework first. Subsequently, the same framework was infiltrated with the gel precursor and cured in-situ to form the final composite electrolyte, after which its mass was measured again. Calculations revealed that approximately 44% of the pores in the porous Na₃Zr₂Si₂PO₁₂ framework were filled with gel, further confirming that the gel electrolyte nearly fully infiltrated the 3D-Na₃Zr₂Si₂PO₁₂ framework. By comparing the mass of the dense ceramic with that of the 3D-Na₃Zr₂Si₂PO₁₂ framework embedded in the gel matrix, the composite gel electrolyte exhibited and ~20% reduction in mass relative to the dense ceramic prepared in this work.

**Supplementary References**

1. Q. Su, S. Huang, J. Liao, D. Song, W. Yuan et al., A flame retardant and flexible gel polymer electrolytes for high temperature lithium metal batteries. J. Electroanal. Chem. **945**, 117712 (2023). <https://doi.org/10.1016/j.jelechem.2023.117712>
2. G. Chen, K. Zhang, Y. Liu, L. Ye, Y. Gao et al., Flame-retardant gel polymer electrolyte and interface for quasi-solid-state sodium ion batteries. Chem. Eng. J. **401**, 126065 (2020). <https://doi.org/10.1016/j.cej.2020.126065>
3. W. Yan, J. Wei, T. Chen, L. Duan, L. Wang et al., Superstretchable, thermostable and ultrahigh-loading lithium–sulfur batteries based on nanostructural gel cathodes and gel electrolytes. Nano Energy **80**, 105510 (2021). <https://doi.org/10.1016/j.nanoen.2020.105510>
4. X. Wu, X. Jie, X. Liang, L. Zhang, J. Wang et al., Polymer/ceramic gel electrolyte with *in situ* interface forming enhances the performance of lithium metal batteries. J. Energy Storage **78**, 110107 (2024). <https://doi.org/10.1016/j.est.2023.110107>
5. Y. Wang, Z. Wei, T. Ji, R. Bai, H. Zhu, Highly ionic conductive, stretchable, and tough ionogel for flexible solid-state supercapacitor. Small **20**(20), 2307019 (2024). <https://doi.org/10.1002/smll.202307019>
6. X. Deng, J. Chen, X. Jia, X. Da, Y. Zhao et al., Highly tough slide-crosslinked gel polymer electrolyte for stable lithium metal batteries. Angew. Chem. Int. Ed. **63**(43), e202410818 (2024). <https://doi.org/10.1002/anie.202410818>
7. J. Zhang, H. Wen, L. Yue, J. Chai, J. Ma et al., *In situ* formation of polysulfonamide supported poly(ethylene glycol) divinyl ether based polymer electrolyte toward monolithic sodium ion batteries. Small **13**(2), 1601530 (2017). <https://doi.org/10.1002/smll.201601530>
8. M.L. Lehmann, G. Yang, D. Gilmer, K.S. Han, E.C. Self et al., Tailored crosslinking of Poly(ethylene oxide) enables mechanical robustness and improved sodium-ion conductivity. Energy Storage Mater. **21**, 85–96 (2019). <https://doi.org/10.1016/j.ensm.2019.06.028>
9. Y. Zhu, Y. Yang, L. Fu, Y. Wu, A porous gel-type composite membrane reinforced by nonwoven: promising polymer electrolyte with high performance for sodium ion batteries. Electrochim. Acta **224**, 405–411 (2017). <https://doi.org/10.1016/j.electacta.2016.12.030>
10. C. Lu, X. Chen, *In situ* synthesized PEO/NBR composite ionogels for high-performance all-solid-state supercapacitors. Chem. Commun. **55**(58), 8470–8473 (2019). <https://doi.org/10.1039/c9cc03401c>
11. Y.-N. Zhou, Z. Xiao, D. Han, L. Yang, J. Zhang et al., Approaching practically accessible and environmentally adaptive sodium metal batteries with high loading cathodes through *in situ* interlock interface. Adv. Funct. Mater. **32**(26), 2111314 (2022). <https://doi.org/10.1002/adfm.202111314>
12. J. Lin, P. Huang, T. Naren, Y. Zhang, L. Zhou et al., Conformally reactive interphase enables excellent kinetics and cyclability in quasi-solid-state sodium metal battery. Energy Storage Mater. **70**, 103495 (2024). <https://doi.org/10.1016/j.ensm.2024.103495>
13. A.A. Bristi, A.J. Samson, A. Sivakumaran, S. Butler, V. Thangadurai, Ionic conductivity, na Plating–Stripping, and battery performance of solid polymer na ion electrolyte based on Poly(vinylidene fluoride) and Poly(vinyl pyrrolidone). ACS Appl. Energy Mater. **5**(7), 8812–8822 (2022). <https://doi.org/10.1021/acsaem.2c01296>
14. T.-H. Park, M.-S. Park, A.-H. Ban, Y.-S. Lee, D.-W. Kim, Nonflammable gel polymer electrolyte with ion-conductive polyester networks for sodium metal cells with excellent cycling stability and enhanced safety. ACS Appl. Energy Mater. **4**(9), 10153–10162 (2021). <https://doi.org/10.1021/acsaem.1c02053>
15. W. Niu, L. Chen, Y. Liu, L.-Z. Fan, All-solid-state sodium batteries enabled by flexible composite electrolytes and plastic-crystal interphase. Chem. Eng. J. **384**, 123233 (2020). <https://doi.org/10.1016/j.cej.2019.123233>
16. Y. Zhang, H. Yuan, L. Shi, H. Lai, X. Wu et al., A biodegradable gel polymer electrolyte based on polydopamine-modified tough polyurethane enabling high-rate sodium batteries. ACS Sustainable Chem. Eng. **12**(8), 3142–3152 (2024). <https://doi.org/10.1021/acssuschemeng.3c07161>
17. X. Zhou, Z. Li, W. Li, X. Li, J. Fu et al., Regulating Na-ion solvation in quasi-solid electrolyte to stabilize Na metal anode. Adv. Funct. Mater. **33**(11), 2212866 (2023). <https://doi.org/10.1002/adfm.202212866>
18. X. Guo, Z. Xie, R. Wang, J. Luo, J. Chen et al., Interface-compatible gel-polymer electrolyte enabled by NaF-solubility-regulation toward all-climate solid-state sodium batteries. Angew. Chem. Int. Ed. **63**(18), e202402245 (2024). <https://doi.org/10.1002/anie.202402245>
19. M. Cheng, T. Qu, J. Zi, Y. Yao, F. Liang et al., A hybrid solid electrolyte for solid-state sodium ion batteries with good cycle performance. Nanotechnology **31**(42), 425401 (2020). <https://doi.org/10.1088/1361-6528/aba059>
20. S. Gao, T. Yang, J. Liu, X. Zhang, X. Zhang et al., Incorporating sodium-conductive polymeric interfacial adhesive with inorganic solid-state electrolytes for quasi-solid-state sodium metal batteries. Small **20**(38), 2401892 (2024). <https://doi.org/10.1002/smll.202401892>
